# Identification of Cytoskeleton-Associated Proteins Essential for Lysosomal Stability and Survival of Human Cancer Cells

**DOI:** 10.1371/journal.pone.0045381

**Published:** 2012-10-11

**Authors:** Line Groth-Pedersen, Sonja Aits, Elisabeth Corcelle-Termeau, Nikolaj H. T. Petersen, Jesper Nylandsted, Marja Jäättelä

**Affiliations:** Cell Death and Metabolism and Center for Genotoxic Stress Research, Danish Cancer Society Research Center, Copenhagen, Denmark; Boston University Medical School, United States of America

## Abstract

Microtubule-disturbing drugs inhibit lysosomal trafficking and induce lysosomal membrane permeabilization followed by cathepsin-dependent cell death. To identify specific trafficking-related proteins that control cell survival and lysosomal stability, we screened a molecular motor siRNA library in human MCF7 breast cancer cells. SiRNAs targeting four kinesins (KIF11/Eg5, KIF20A, KIF21A, KIF25), myosin 1G (MYO1G), myosin heavy chain 1 (MYH1) and tropomyosin 2 (TPM2) were identified as effective inducers of non-apoptotic cell death. The cell death induced by KIF11, KIF21A, KIF25, MYH1 or TPM2 siRNAs was preceded by lysosomal membrane permeabilization, and all identified siRNAs induced several changes in the endo-lysosomal compartment, *i.e.* increased lysosomal volume (KIF11, KIF20A, KIF25, MYO1G, MYH1), increased cysteine cathepsin activity (KIF20A, KIF25), altered lysosomal localization (KIF25, MYH1, TPM2), increased dextran accumulation (KIF20A), or reduced autophagic flux (MYO1G, MYH1). Importantly, all seven siRNAs also killed human cervix cancer (HeLa) and osteosarcoma (U-2-OS) cells and sensitized cancer cells to other lysosome-destabilizing treatments, *i.e.* photo-oxidation, siramesine, etoposide or cisplatin. Similarly to KIF11 siRNA, the KIF11 inhibitor monastrol induced lysosomal membrane permeabilization and sensitized several cancer cell lines to siramesine. While KIF11 inhibitors are under clinical development as mitotic blockers, our data reveal a new function for KIF11 in controlling lysosomal stability and introduce six other molecular motors as putative cancer drug targets.

## Introduction

Lysosomes are acidic vesicles containing numerous hydrolases, which degrade organelles and macromolecules delivered to them by autophagy, endocytosis and phagocytosis [1]. Enhanced lysosomal synthesis, trafficking and extracellular release of lysosomal proteases (cathepsins) are important hallmarks of cancer and are associated with the metastatic and invasive capacity of cancer cells [2], [3], [4]. Interestingly, these transformation-associated changes sensitize cancer cells to the lysosomal cell death pathway [5], a form of programmed cell death that can take over when apoptosis is inhibited, as is the case in many cancers [6]. Lysosomal cell death is characterized by lysosomal permeabilization and subsequent translocation of cathepsins into the cytosol where they activate apoptosis or carry out death without caspase activation [3].

Among the cancer drugs that activate lysosomal cell death are microtubule-destabilizing and -stabilizing drugs (*e.g.* vinca alkaloids and taxanes), which inhibit lysosomal trafficking and induce an expansion of the lysosomal compartment followed by lysosomal rupture and cathepsin-dependent cell death [7], [8]. Unfortunately, such a severe cytoskeletal disturbance also affects vital processes in healthy cells leading to toxicity in patients [9]. A more specific targeting of lysosomal trafficking might thus improve therapy considerably.

Cytoskeleton dynamics and intracellular transport of vesicles, organelles and macromolecules along the microtubule and actin cytoskeletons depend on molecular motor proteins. They can be divided into kinesins, dyneins and myosins, all of which have been implicated in lysosome trafficking [10], [11], [12]. Additionally, numerous accessory proteins regulate the function of motor proteins [13], [14], [15]. Kinesins and dyneins, which move along microtubules, transport a variety of cargo and help create the mitotic spindle. The 44 known human kinesins move predominantly towards plus ends of microtubules in the periphery of the cell (anterograde transport) [13]. In contrast, the two known human cargo-transporting dynein heavy chains, which form functioning motor protein complexes with several accessory proteins, move towards minus ends of microtubules in the perinuclear area of the cell (retrograde transport) [14]. In addition, the human genome encodes for fourteen axonemal dyneins responsible for the sliding of microtubules that causes the beating of cilia and flagella. Myosins, of which humans have ∼40, bind to actin filaments that are concentrated beneath the plasma membrane. They are especially important for short-range transport during endocytosis and exocytosis. Myosins also generate mechanical force for muscle contraction, cell migration and cytokinesis [15]. Other actin-binding proteins such as tropomyosins, which affect actin dynamicity and stability [16], modulate myosin function.

To identify molecular motors and related proteins required for cancer cell survival, we screened an siRNA library targeting 136 molecular motors and related proteins for siRNAs that reduce the viability of MCF7 cells. The seven proteins identified were then characterized for their role in cell death, cell cycle, cytoskeleton structure, autophagy, lysosomal function and lysosomal integrity. Remarkably, depletion of all identified proteins triggered non-apoptotic cell death that was preceded by dramatic changes in lysosomal stability and function.

## Results

### Identification of cytoskeleton-associated proteins whose depletion induces non-apoptotic cancer cell death

**Figure 1:**
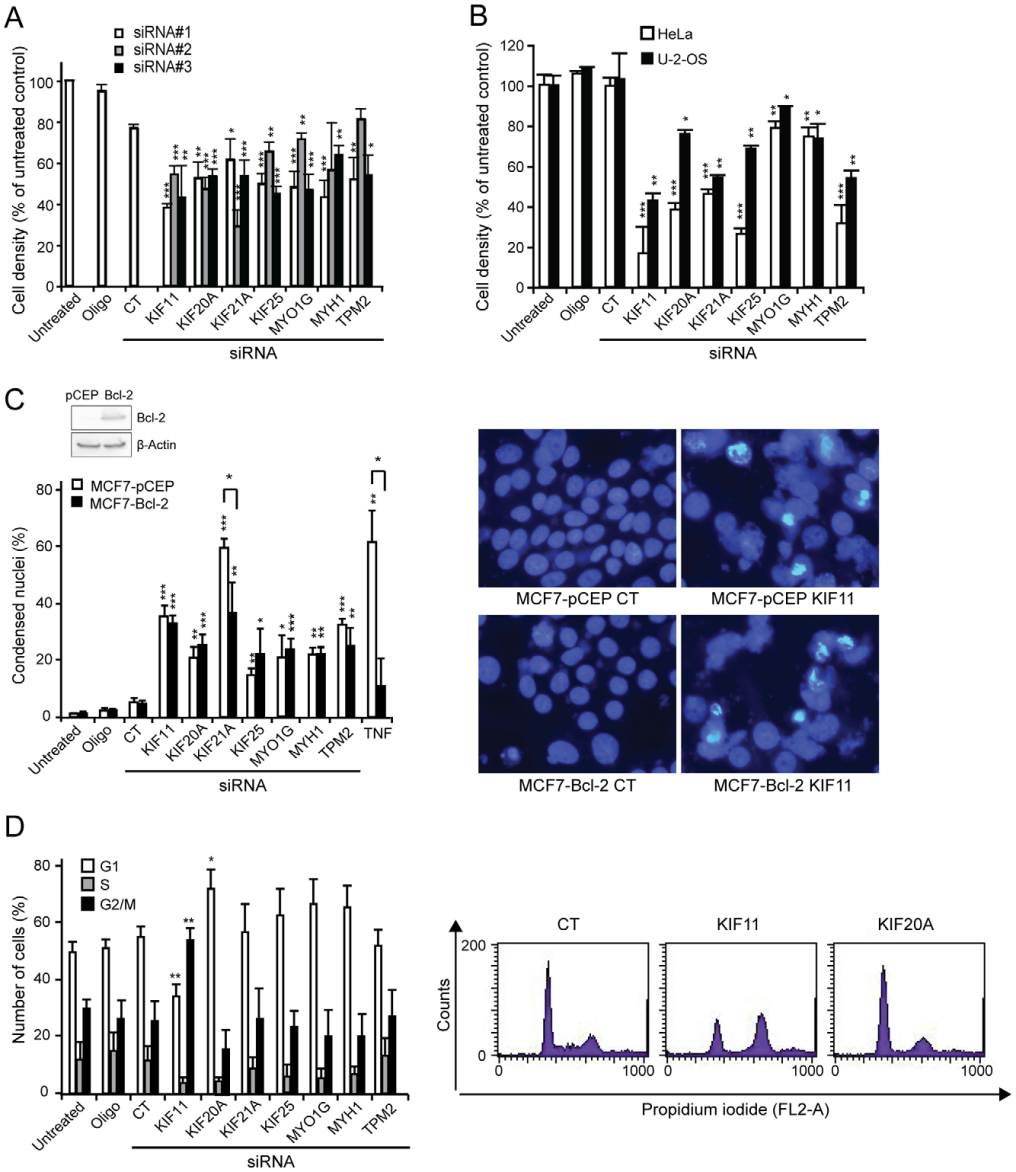
Identification of cytoskeleton-associated proteins whose depletion induces non-apoptotic cancer cell death. (A, B) MCF7 (A), HeLa (B) and U-2-OS (B) cells were left untreated, treated with Oligofectamine (Oligo) or transfected with control siRNA (CT) or three independent siRNAs against the indicated targets individually (8 nM; A) or in pools (3×6.67 nM; B). Cell density was measured after 72 h by the MTT reduction assay. (C) MCF7-Bcl-2 or MCF7-pCEP (control) cells were transfected as in (B). *Left bottom*, After 96 h, cell death was determined by counting Hoechst 33342-stained cells with condensed nuclei (three random fields of 100 cells). TNF (20 ng/ml, 24 h) served as a positive control for Bcl-2 sensitive apoptotic cell death. *Left top*, Western blot confirming overexpression of Bcl-2 in untreated MCF7-Bcl-2 cells. *Right*, Examples of images of Hoechst 33342-stained nuclei of MCF7-pCEP and MCF7-Bcl-2 cells 96 h after transfection with indicated siRNAs. (D) MCF7 cells were treated as in (B). *Left*, After 60 h, DNA was stained with propidium iodide and cell cycle distribution analyzed by flow cytometry (FL-2A). *Right*, Examples of histograms showing cell cycle distribution of cells 60 h after transfection with indicated siRNAs. Values represent means + SD of three independent experiments (A, C, D) or triplicates in one representative experiment (B, n = 3). *P<0.05, **P<0.01, ***P<0.001, vs. control siRNA-transfected cells or as indicated (C).

Cytoskeleton-disrupting drugs are potent inducers of lysosomal cell death [7], [8]. To identify cytoskeleton-regulating proteins necessary for cancer cell survival, we screened an Ambion Silencer® Molecular Motor Library (Table S1) for toxic effects on MCF7 breast cancer cells using the MTT reduction assay. Proteins were considered candidates if ≥2/3 siRNAs reduced cell density by >40% in three independent experiments. Four kinesin family members (KIF11, KIF20A, KIF21A, KIF25), two myosins (MYO1G and MYH1) and tropomyosin 2 (TPM2) fulfilled these criteria (Fig. 1A) and were further analyzed after confirming knockdown by the siRNAs (Fig. S1).

For subsequent experiments the three siRNAs for each target were pooled if not otherwise indicated. As in MCF7 cells, the depletion of the identified proteins reduced the density of HeLa cervix carcinoma and U-2-OS osteosarcoma cells significantly even though the pattern differed somewhat from that observed in MCF7 cells (Fig. 1A, B). This result was confirmed using single siRNAs in U-2-OS cells (data not shown). Next, we examined whether the observed cell death was Bcl-2-sensitive (apoptotic) by transfecting Bcl-2-overexpressing and vector-transfected MCF7 cells [17] with the siRNAs and quantifying death-associated chromatin condensation after 96 h. The seven siRNAs caused chromatin condensation in 20–60% of the cells. Bcl-2 inhibited chromatin condensation only after tumor necrosis factor (TNF) treatment (a control for apoptotic cell death), and partially in KIF21A siRNA-transfected cells (Fig. 1C). Notably, KIF21A siRNA still induced nuclear condensation in ∼40% of the Bcl-2-overexpressing cells (Fig. 1C). Similar results were obtained with single siRNAs (data not shown).

KIF11 and KIF20A are known to regulate mitotic spindle formation and cytokinesis, respectively [18], [19]. KIF11 depletion arrested the cells in G2/M phase, as expected, whereas KIF20A siRNA-transfected cells accumulated in G1 phase (Fig. 1D). The other siRNAs caused no significant changes in cell cycle distribution (Fig. 1D).

### Effect of the identified siRNAs on lysosomes and cytoskeleton

**Figure 2:**
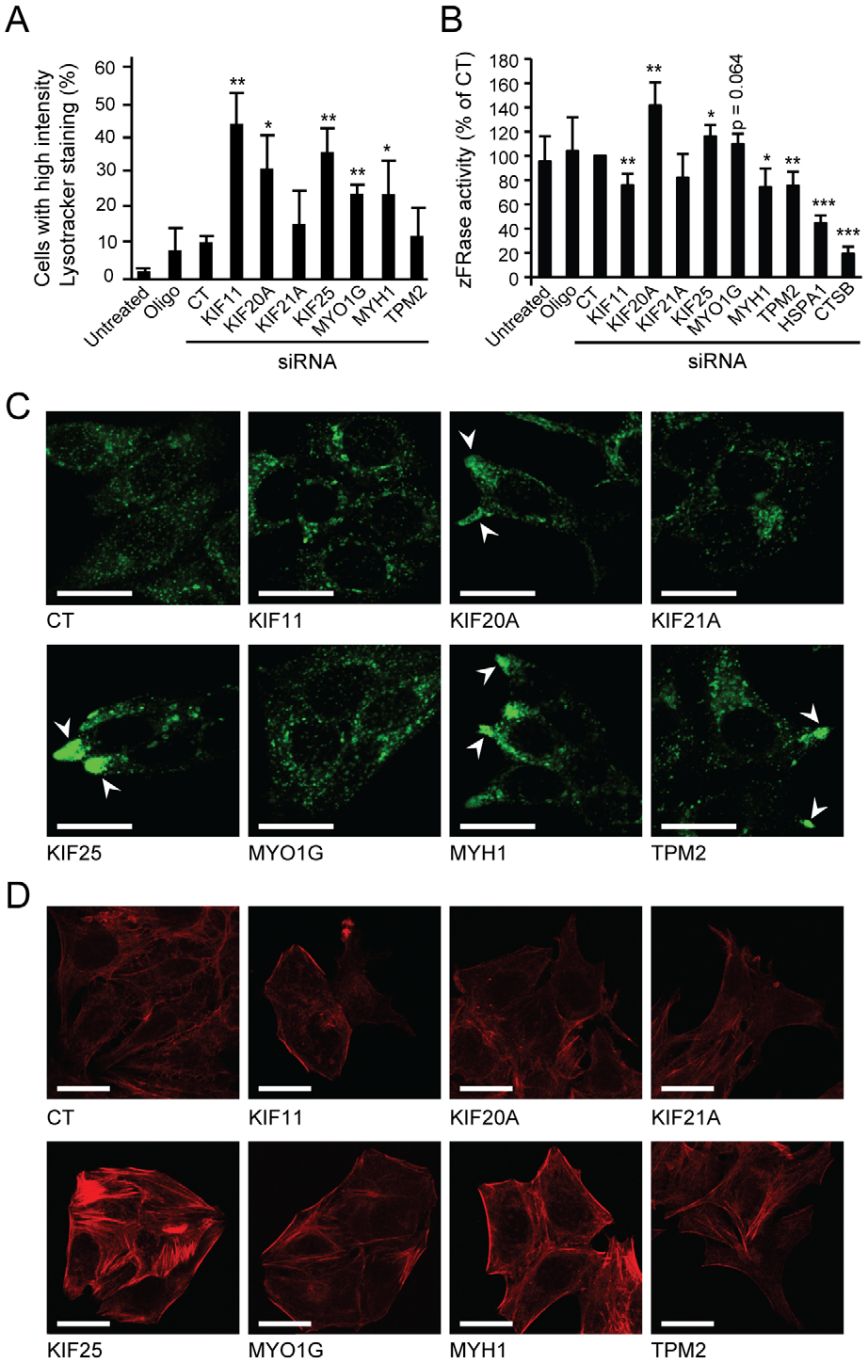
Effect of the identified siRNAs on lysosomes and cytoskeleton. (A–D) MCF7 cells were left untreated, treated with Oligofectamine (Oligo) or transfected with control siRNA (CT) or indicated siRNA pools. (A) After 60 h, cells with enlarged acidic compartment (late endosomes and lysosomes) were identified by flow cytometry (FL-2A) of LysoTracker Red-stained cells. The threshold for high intensity staining was defined so that 90% of control siRNA-transfected cells were below. (B) After 72 h, total cysteine cathepsin activity (zFR-AFC cleavage) was determined. HSPA1 and CTSB siRNAs served as internal controls. (C, D) After 60 h, cells were stained for Lamp-2 (C) or F-actin (D) and analyzed by confocal microscopy. Representative images are shown. *Arrows*, aggregation of lysosomal structures in cell protrusions/periphery. *Bars*, 20 µm. Values represent means + SD of a minimum of three independent experiments. *P<0.05, **P<0.01, ***P<0.001, vs. control siRNA-transfected cells.

Since non-apoptotic cell death can result from lysosomal damage, we next studied the effect of the identified siRNAs on lysosomes in MCF7 cells. KIF11, KIF20A, KIF25, MYO1G and MYH1 siRNAs significantly increased the proportion of cells with an enlarged endo-lysosomal (acidic) compartment (Fig. 2A), and in cells depleted for KIF20A, KIF25 or MYO1G this increase was associated with increased lysosomal protease activity (Fig. 2B). On the contrary, KIF11, MYH1 and TPM2 siRNAs reduced cathepsin activity possibly due to lysosomal membrane permeabilization (see below). Lysosomes were dispersed throughout the cytoplasm in cells transfected with control, KIF11, KIF21A or MYO1G siRNAs (Fig. 2C). On the contrary, KIF20A-depleted cells displayed long protrusions that were often densely populated by lysosomes, and KIF25, TPM2 and MYH1 siRNAs caused peripheral lysosomal aggregation (Fig. 2C). Similar lysosomal distribution was observed upon transfection with all three single siRNAs targeting KIF20A, KIF25 and MYH1 and siRNAs 1 and 3 targeting TPM2 (data not shown).

Next, we examined whether the altered lysosomal localization was associated with changes in the actin or microtubule cytoskeleton, which are both involved in lysosomal trafficking [20]. Depletion of KIF25 and MYH1 dramatically increased F-actin levels and stress fibers which may contribute to the lysosomal relocalization (Fig. 2D). A smaller increase in stress fibers was observed upon treatment with MYO1G and TPM2 siRNAs, whereas no changes were seen with the other siRNAs (Fig. 2D). None of the identified siRNAs had detectable effects on microtubules as visualized by α-tubulin staining (data not shown).

### Effect of the identified siRNAs on autophagy and dextran accumulation

**Figure 3:**
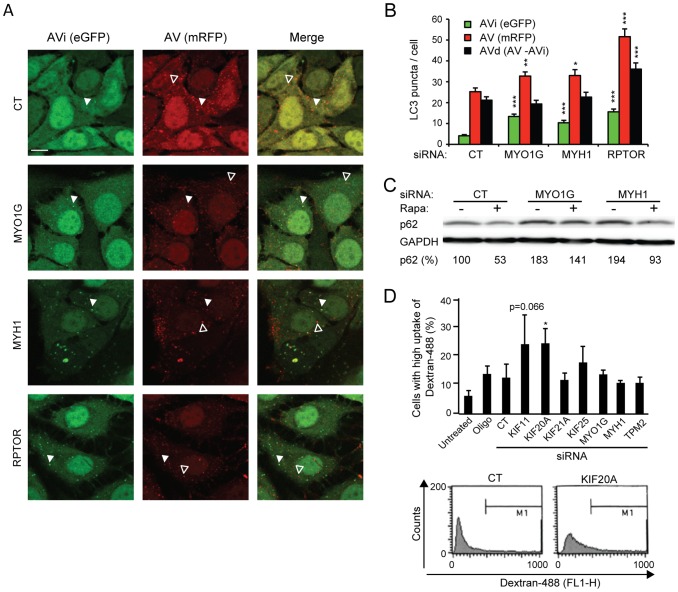
Effect of the identified siRNAs on autophagy and dextran uptake. (A–D) tfLC3-MCF7 cells (A, B) or MCF7 cells (C, D) were left untreated, treated with Oligofectamine (Oligo) or transfected with control siRNA (CT) or indicated siRNA pools (3×6.67 nM). (A, B) After 48 h, tfLC3-MCF7 cells were analyzed by confocal microscopy. Representative images (A; *Bars*, 10 µm) and quantification of puncta (B) are shown. Raptor siRNA (RPTOR) served as a control for increased autophagic flux. Closed arrows indicate AVd, open arrows indicate AVi. (C) After 60 h, the level of p62/SQSTM1 (p62), which is degraded by autophagy, was examined by Western blot. Rapamycin (20 nM, 4 h) was used to induce autophagy. Numbers represent p62 levels as percentage of the level in untreated control siRNA-transfected cells. (D) *Top*, After 60 h, MCF7 cells were treated with 100 µg/ml Alexa Fluor 488-dextran (dextran-488) for 1 h and analyzed by flow cytometry (FL1-H). The threshold for high intensity staining was defined so that 88% of control siRNA-transfected cells were below. *Bottom*, Example histograms showing dextran-488 content of cells transfected with control or KIF20A siRNA. M1 = gate for high intensity staining. Values represent means + SEM of 20 cells in one representative experiment (B, n = 3) or means + SD of three independent experiments (D). *P<0.05, **P<0.01, ***P<0.001, vs. control siRNA-transfected cells.

Lysosomes receive their cargo mainly through autophagy and endocytosis. To test the effect of the identified siRNAs on autophagy, we used MCF7 cells expressing tfLC3, a pH-sensitive tandem fluorescent protein consisting of monomeric red fluorescent protein (mRFP), enhanced green fluorescent protein (eGFP) and microtubule-associated protein 1 light chain 3 (LC3) [21]. In initial autophagic vacuoles (AVi) tfLC3 emits green and red fluorescence whereas in degradative autophagic vacuoles (AVd) it fluoresces only red since eGFP fluorescence is lost in acidic amphisomes and autolysosomes. As reported previously [22], depletion of raptor, a component of the mammalian target of rapamycin complex 1 that normally blocks autophagy, increased the number of both AVi and AVd indicative of increased autophagic flux (Fig. 3A, B). In contrast, MYO1G and MYH1 siRNA pools as well as all three single siRNAs targeting MYH1 and siRNAs 1 and 3 targeting MYO1G increased AVi but not AVd. SiRNAs targeting the other five candidates had no apparent effects in this assay (Fig. 3A, B and data not shown). The ability of MYO1G and MYH1 siRNAs to inhibit autophagic flux was also indicated by an increase in p62/sequestesome (p62/SQSTM1, a protein effectively degraded by autophagy) levels and reduced ability of an autophagy inducer, rapamycin, to reduce p62/SQSTM1 levels after siRNA treatments (Fig. 3C).

Next, we examined the effect of the identified siRNAs on the uptake of 10 kDa Alexa Flour 488-dextran by flow cytometry. KIF20A depletion increased the accumulation of dextran significantly while KIF11 siRNA caused a slight (p = 0.066) increase. The other five siRNAs had no effect in this assay (Fig. 3D). It should be noted that this assay cannot distinguish between increased endocytosis and decreased exocytosis.

### Reduction of lysosomal stability by the identified siRNAs and monastrol

**Figure 4:**
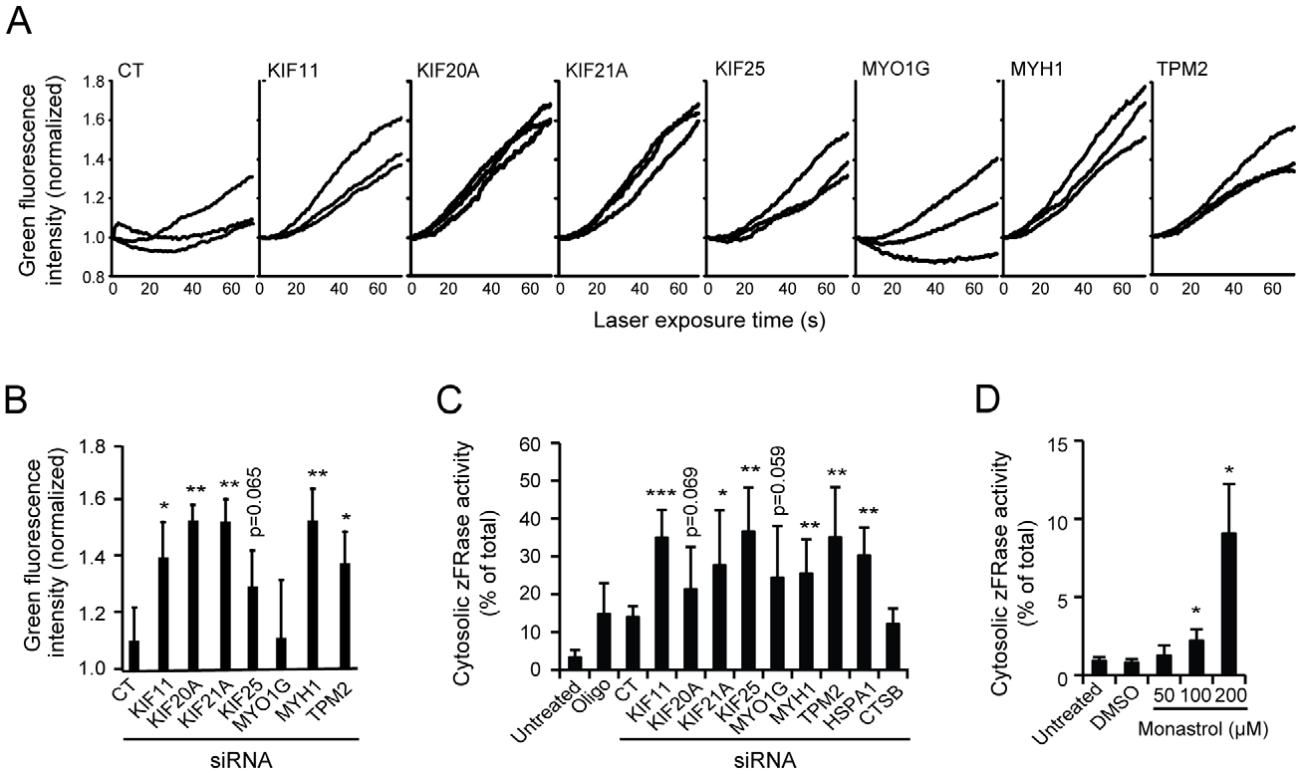
Reduction of lysosomal stability by the identified siRNAs and monastrol. (A–C) MCF7 cells were left untreated, treated with Oligofectamine (Oligo) or transfected with control siRNA (CT) or indicated siRNA pools (3×6.67 nM). (A and B) After 60 h, cells were treated with acridine orange and analyzed by live cell imaging to measure the loss of lysosomal integrity (increased green fluorescence) upon laser treatment. A miminum of 25 cells from pre-defined areas was examined for each experiment. Three independent experiments are shown in A and values in B represent means + SD of these experiments at the 60 sec time point. (C) After 72 h, cytosolic and total cysteine cathepsin activities were measured by analyzing the cleavage of zFR-AFC. The activities in cytosolic extracts are shown as percentages of the activities in the corresponding total extracts. HSPA1 and CTSB siRNAs served as controls for the induction of lysosomal leakage and transfection efficacy, respectively. (D) Cytosolic cysteine cathepsin activities in MCF7 cells left untreated or treated with vehicle (2% dimethyl sulfoxide, DMSO) or indicated concentrations of monastrol for 72 h were determined as in (C). Values represent means + SD of five (C) or three (D) independent experiments. *P<0.05, **P<0.01, ***P<0.001, vs. control siRNA-transfected (B, C) or vehicle-treated cells (D).

The non-apoptotic cell death and numerous lysosomal changes observed above prompted us to study the effect of the identified siRNAs on lysosomal stability. First, we measured the ability of lysosomes to retain acridine orange, a metachromatic basic dye, when challenged with blue light [23]. KIF11, KIF20A, KIF21A, MYH1 and TPM2 siRNAs sensitized MCF7 cells significantly to photo-oxidation-induced lysosomal leakage and KIF25 siRNA showed a similar tendency 60 h after the transfection (Fig. 4A, B). When analyzed after 72 h, all siRNAs had induced lysosomal leakage (appearance of lysosomal proteases in the cytosol), even though the effect of KIF20A and MYO1G siRNAs did not quite reach statistical significance (Fig. 4C). Notably, treatment of MCF7 cells with monastrol, a well-characterized small molecule inhibitor of KIF11 [24], also induced lysosomal membrane permeabilization (Fig. 4D). Thus, the depletion of each of the seven proteins as well as monastrol treatment results in lysosomal destabilization.

### Sensitization to lysosome-disrupting drugs by the identified siRNAs and monastrol

**Figure 5:**
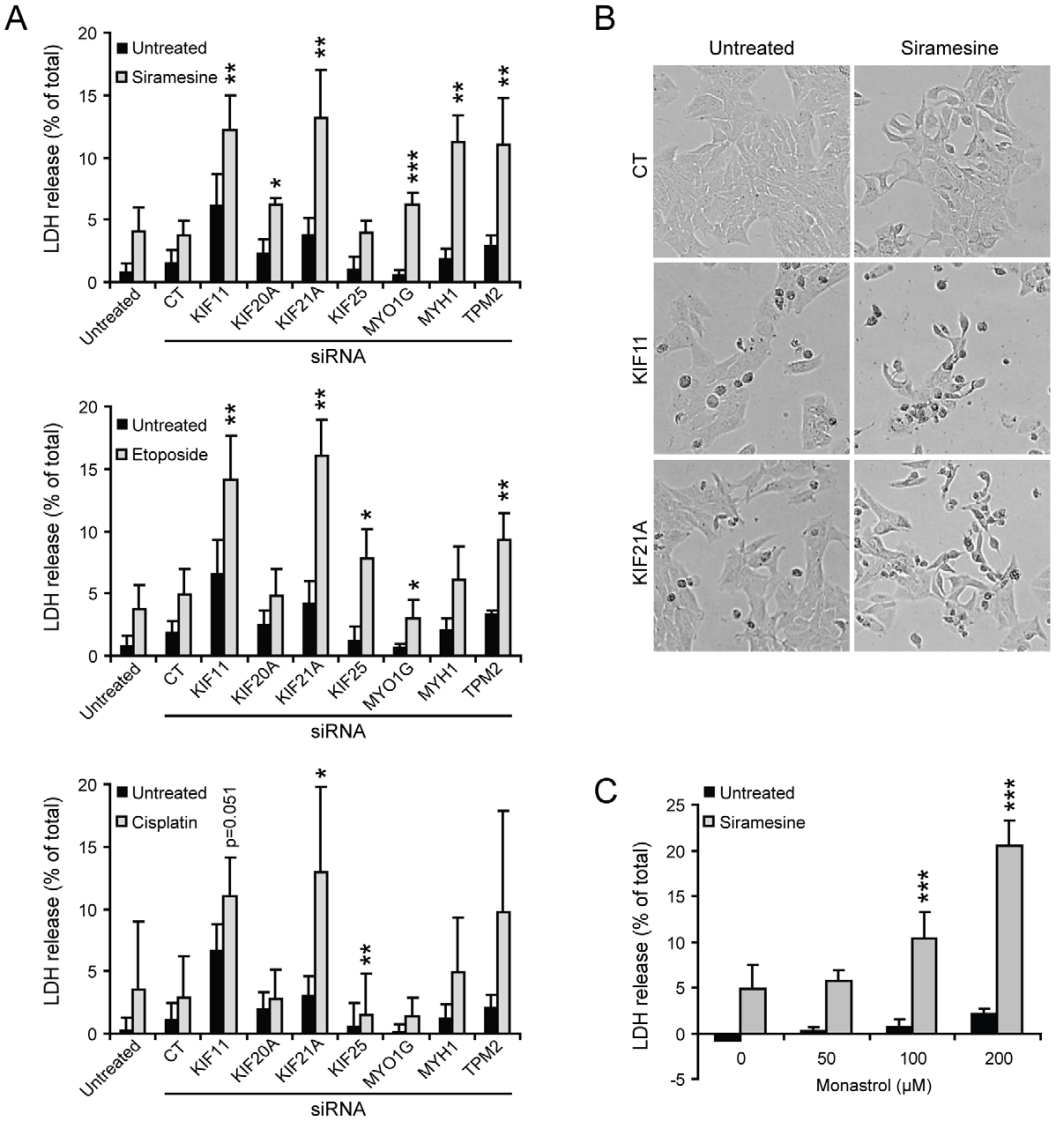
Sensitization to lysosome-disrupting drugs by the identified siRNAs and monastrol. (A, B) MCF7 cells were left untreated, treated with Oligofectamine (Oligo) or transfected with control siRNA or indicated siRNA pools (3×6.67 nM). After 48 h, cells were left untreated or treated with 2 µM siramesine (*top*), 50 µM etoposide (*middle*) or 10 µM cisplatin (*bottom*) for additional 48 h before light microscopy pictures were taken (representative images in B) and cell death was quantified by the LDH release assay (A). (C) MCF7 cells were left untreated or treated with 2 µM siramesine together with indicated concentrations of monastrol for 72 h and cell death was quantified by the LDH release assay. Values represent means + SD of a minimum of three independent experiments. *P<0.05, **P<0.01, ***P<0.001, vs. control siRNA-transfected cells (A) or cells treated with 2 µM siramesine alone (C).

Since the siRNAs destabilized the lysosomes, we examined whether they would also sensitize cells to lysosome-disrupting drugs. For this purpose, MCF7 cells were transfected with siRNAs for 48 h and then treated for an additional 48 h with siramesine, etoposide or cisplatin, all of which are capable of causing lysosomal cell death [5], [25]. All siRNAs except KIF25 siRNA sensitized cells to siramesine with the strongest effect observed for KIF11 and KIF21A siRNAs (Fig. 5A, B). For KIF11, this was confirmed using the three single siRNAs (data not shown). Sensitization to etoposide was seen with KIF11, KIF21A, KIF25, MYH1 and TPM2 siRNAs (Fig. 5A, B). KIF20A siRNA had no effect, while MYO1G siRNA reduced cell death in response to etoposide, possibly due to its ability to inhibit autophagy, which may contribute to etoposide-induced death [26]. In addition, KIF11, KIF21, MYH1 and TPM2 siRNAs enhanced cisplatin-induced cell death but due to variations between experiments the effect was only significant for KIF21A siRNA. Furthermore, combining monastrol and siramesine resulted in synergistic induction of cell death in MCF7, HeLa, U-2-OS and DU-145 cells (Fig. 5C, S2). Thus, all siRNAs sensitized cancer cells to one or several lysosome-disrupting drugs with the strongest effects observed in cells lacking KIF11 or KIF21A.

## Discussion

In this study, we identified KIF11, KIF20A, KIF21, KIF25, MYO1G, MYH1 and TPM2 as proteins whose depletion causes growth inhibition and non-apoptotic cell death in cancer cells (Table 1). To our knowledge, this study is the first one to identify KIF21A, KIF25, MYO1G, MYH1 and TPM2 as proteins essential for cancer cell survival, whereas others have earlier reported cell death upon depletion of KIF11 [27], [28], [29] and KIF20A [30] in other cancer cell lines. Similarly to the findings in our previous study showing that the depletion of KIF5B is more toxic to HeLa cells than to MCF7 cells [31], we observed some differences in the sensitivities of the different cancer cell lines to the identified siRNAs. This may be due to differences in expression levels of the target genes or related genes with redundant functions.

**Table 1:** Summary of cellular changes induced by the depletion of survival-associated motor proteins.

siRNA	Cell density	Cell death	CC^1^ arrest	VAC^2^	zFRase activity	Lysosomal localization	Stress fibers	Lysosomal leakage	Lysosomal leakage, PO^3^	Dextran uptake	Autoph. flux	Sensitivity to drugs		
	72 h	96 h	60 h	60 h	72 h	60 h	60 h	72 h	60 h	60 h	48 h	48+48 h		
												Sira	Eto	Cis
KIF11	−4	+	G2/M	+	**−**	±	±	+	+	(+)	±	+	+	(+)
KIF20A	−	+	G1	+	+	Protrusions	±	(+)	+	+	±	+	±	±
KIF21A^5^	−	+	±	±	±	±	±	+	+	±	±	+	+	+
KIF25	−	+	±	+	+	Peripheral	+	+	(+)	±	±	±	+	±
MYO1G	−	+	±	+	(+)	±	+	(+)	±	±	−	+	−	±
MYH1	−	+	±	+	−	Peripheral	+	+	+	±	−	+	±	±
TPM2	−	+	±	±	−	Peripheral	+	+	+	±	±	+	+	±

1 CC, cell cycle.

2 VAC, volume of the acidic compartment (late endosomes and lysosomes).

3 photo-oxidation-induced lysosomal leakage.

4 −, decrease, *P*<0.05; +, increase, *P*<0.05; ±, no change; () 0.05<*P*<0.10.

5 It should be noted that these data might be affected by cell death that starts already 50 hours after the transfection with KIF21A siRNAs.

Notably, ectopic expression of Bcl-2 failed to rescue MCF7 cells from the cytotoxicity induced by all identified siRNAs except KIF21A siRNA. Even in KIF21A-depleted cells, ectopic Bcl-2 reduced cell death only partially from 60 to 40%. The insensitivity to Bcl-2 suggested the involvement of alternative cell death mechanisms rather than classical apoptosis. This notion was strongly supported by the subsequent observation that the depletion of all seven proteins caused some degree of lysosomal destabilization, a hallmark of the lysosomal cell death pathway [3], [32]. It is, however, not immediately obvious how depletion of the identified proteins leads to lysosomal disruption.

Of the identified kinesins, KIF11, also called kinesin spindle protein or Eg5, has been studied most extensively, especially in the context of cancer [33]. KIF11 forms a homotetramer that is responsible for spindle formation during mitosis [18]. Accordingly and consistent with other studies [28], [29], KIF11 depletion arrested MCF7 cells in the G2/M cell cycle phase. KIF11 inhibition has also been reported to kill human ovarian carcinoma and leukemia cells via the intrinsic apoptotic pathway in a Bcl-2-sensitive manner [28], [34]. In contrast, KIF11 siRNA caused Bcl-2-insensitive non-apoptotic death in MCF7 cells which likely resulted from the destabilization of the lysosomes and the subsequent release of cysteine cathepsins into the cytosol. KIF11 inhibition may trigger the lysosomal cell death pathway also in other cell types since lysosome-stabilizing Hsp70 protects myeloma cells against cytotoxicity induced by dimethylenastron, a pharmacological inhibitor of KIF11 [29].

Similarly to KIF11, depletion of KIF21A caused excessive lysosomal permeabilization and cell death. It should be noted that the cell death induced by KIF21A depletion started already ∼50 h after transfection and might thus have affected other measurements of lysosomal function in this study (Table 1). KIF21A binds to the guanine nucleotide-exchange factor BIG1 [35], which helps to maintain the organization of the Golgi apparatus [36]. Thus, KIF21A depletion might affect trafficking of lysosomal components from the Golgi apparatus to the endo-lysosomal compartment thereby causing lysosomal dysfunction. Otherwise, practically nothing is known about KIF21A and our results strongly encourage further examination of its role in normal and cancer cells.

The third kinesin identified in our screen, KIF20A (also called Rabkinesin-6, RAB6KIFL or MKlp2) has been reported to be essential for cytokinesis in HeLa cells in which its inhibition results in the formation of multinucleated cells [19], [37], and for the survival of pancreatic cancer cells by a mechanism not involving blockage of cytokinesis [30]. Similarly to pancreatic cancer cells, KIF20A-depleted MCF7 cells did not arrest in mitosis or display a multinucleated phenotype suggesting that other kinesins may have taken over its mitotic function in these cells. Instead, KIF20A depletion resulted in the accumulation of MCF7 cells in the G1 phase of the cell cycle and caused lysosomal cell death. The cell death was preceded by increased lysosomal volume, cysteine cathepsin activity and dextran accumulation and destabilization of lysosomal membranes. The observed effects on the endo-lysosomal compartment may be related to another previously reported function of KIF20A, namely its involvement in the trafficking of Golgi-related vesicles to the plasma membrane through an interaction with Rab6 [30], [38].

Depletion of the last identified kinesin, KIF25, caused peripheral lysosomal aggregation and an increase in lysosomal volume, a phenotype resembling that caused by microtubule-disturbing drugs [7]. Deregulated trafficking and increased lysosomal volume may have contributed to the lysosomal permeabilization as enlarged lysosomes are prone to disruption [39]. KIF25 depletion also caused formation of actin stress fibers, which may be due to altered Rho signaling as previously observed upon microtubule destabilization [40]. These first clues to the KIF25 function in lysosomal trafficking and cancer biology warrant a closer study of this largely unknown member of the kinesin family.

In addition to the microtubule-interacting kinesins, we identified three actin-binding proteins, MYH1, MYO1G and TPM2, as essential proteins for cancer cell survival. MYH1, also called Myosin heavy chain 2× (MyHC-2X), is part of the sarcomere in fast skeletal muscle fibers [41]. Its functions in non-muscle cells are practically unknown but it may help organize actin fibers and thereby affect actin-dependent trafficking or organelle anchorage. In accordance with this, MYH1-depleted MCF7 cells showed an increase in actin stress fibers and peripheral lysosomal aggregation accompanied by an expanded lysosomal compartment and lysosomal permeabilization. In addition, MYH1 depletion caused inhibition of autophagic degradation and accumulation of initial autophagic vacuoles indicative of defective autophagosome-lysosome fusion, which may be due to the misplacement of lysosomes.

The second identified myosin, MYO1G, is enriched at the plasma membrane of hematopoietic cells where it has been suggested to enhance cellular elasticity [42], [43]. As other class I myosins [15], MYO1G may also be involved in vesicle trafficking. However, neither lysosomal localization nor dextran accumulation changed in MYO1G-depleted cells, and the other lysosomal effects were milder than after depletion of the other identified hits. MYO1G depletion had, however, a strong inhibitory effect on autophagic flux, which might result from the observed changes in actin fibers. Recently, MYH9/NMHC-IIA was found to be involved in autophagosome formation during starvation [44], and our results indicate that the role of additional myosins, especially MYH1 and MYO1G, in autophagy should be investigated further.

The only non-motor protein identified in our screen was TPM2, which forms filaments along actin fibers and controls muscle contraction by blocking actin-myosin interaction. In non-muscle cells, TPM2 and other tropomyosins are believed to stabilize actin filaments and regulate actin functions including cell motility and organelle and vesicle transport [16]. TPM2 depletion caused peripheral lysosomal aggregation indicating that TPM2 may, indeed, function in actin-dependent lysosomal trafficking. This is consistent with data showing that microinjection of TPM1 and TPM2 antibodies inhibits the transport of intracellular granules [45].

Deleterious lysosomal changes observed upon depletion of KIF25, TPM2 and MYH1 may be linked to their apparent function in lysosomal trafficking but it remains less clear how down-regulation of the other proteins disturbed lysosomes. It is possible that their depletion had subtle effects on lysosomal trafficking, such as changes in the short-range trafficking of lysosomes or trafficking of a lysosome subpopulation, which were not detectable with the used methods. Alternatively, the transport of lipids or proteins that promote lysosomal integrity, such as lysosomal membrane proteins, Hsp70 and acid sphingomyelinase [5], [23], might have been altered. More indirectly, their depletion may cause cytoskeletal changes that damage other cellular organelles and thereby activate signaling cascades that trigger lysosomal permeabilization.

The identified proteins may be suitable targets for cancer therapy as cancer cells are sensitized to lysosomal cell death [4], [5]. Several inhibitors of KIF11, which is upregulated in a wide range of cancers (Oncomine, http://www.oncomine.org), are already in clinical trials as anti-cancer drugs [9], and a KIF20A inhibitor has recently been identified [46]. These inhibitors were developed as mitotic blockers but our results indicate that their anti-cancer activity may as well result from lysosomal disruption. We also found that depletion of the seven hits enhanced the toxicity of photo-oxidation and of the lysosome-disrupting drugs siramesine, etoposide and cisplatin. Strong synergism with all drugs was observed after depletion of KIF11, KIF21A and TPM2 whereas downregulation of the other proteins was synergistic only with some of the drugs, possibly reflecting differences in the mechanism of lysosomal disruption or drug uptake. Consequently, combining motor protein inhibition with other lysosome-disrupting treatments appears to be a promising strategy for cancer therapy. This should especially be tested for the already available KIF11 inhibitors, which have only modest anti-cancer effects as single agents [9].

In addition to the cancer connections studied here, our results provide clues to the etiology of rare genetic disorders caused by mutations in KIF21A and TPM2. KIF21A mutations are found in patients with congenital fibrosis of extraocular muscles, a strabismus syndrome associated with defects of the oculomotor nerve [47], and TPM2 mutations are associated with myophathies [48]. Interestingly, both strabismus and myopathies occur also in patients suffering from lysosomal storage disorders where the absence/malfunctioning of lysosomal proteins causes lysosomal dysfunction [49], [50]. Thus, some of the symptoms associated with KIF21A or TPM2 mutations might be caused by lysosomal dysfunction.

In conclusion, the present findings increase our understanding of the functions of seven trafficking-related proteins, whose functions have hitherto been poorly understood, and identify these proteins as potential drug targets for anti-cancer therapy.

## Materials and Methods

### Cells and treatments

The MCF7 cell line used here is a subclone of MCF7 human breast adenocarcinoma cells selected for high TNF sensitivity [51]. MCF7-pCEP and MCF7-Bcl-2 cells are MCF7 clones stably transfected with empty vector or Bcl-2 [17]. HeLa, U-2-OS and DU-145 cells were obtained from American Type Culture Collection. Cells were cultured as described [7].

Human TNF and siramesine were kindly provided by Dr. Anthony Cerami (Kenneth Warren Laboratories, Tarrytown, NY, USA) and Dr. Christiane Volbracht (H. Lundbeck A/S, Valby, Denmark), respectively. Other drugs were from Sigma-Aldrich.

### RNA interference

An Ambion Silencer® Molecular Motor library (Table S1) was used to deplete molecular motors. Cells were transfected with single (8 nM) or pooled siRNAs (3×6.7 nM) using Oligofectamine (Invitrogen). CTSB siRNA (described previously [52]), Hsp70 (HSPA1) siRNA and non-targeting siRNA (both described previously [53]) as well as AllStar Negative Control siRNA (QIAGEN) were used as controls. Knockdown was verified by reverse transcription-PCR (RT-PCR) or Western blot (see below).

### Cell density and cell death

Cell density/viability was quantified by the 3-(4,5-dimethylthiazole-2-yl)-2,5-diphenyltetrazolium bromide (MTT) reduction assay and cell death by the lactate dehydrogenase (LDH) release assay (Cytotoxicity Detection kit, Roche Applied Science) or by counting condensed nuclei in Hoechst 33342-stained cells (Sigma-Aldrich) in an OLYMPUS IX71 microscope (UV channel) as described previously [5], [7]. Bright-field pictures were taken with the same microscope.

### Flow cytometry

Flow cytometry analysis was performed in a FACSCalibur (Becton Dickinson) essentially as described previously [7]. The probes used included propidium iodide for DNA content, LysoTracker® Red DND-99 (Molecular Probes) for the volume of the acidic compartment, and Alexa Fluor 488-dextran (Molecular Probes) for endocytosis/exocytosis.

### Cathepsin activity

Cysteine cathepsin activity (zFRase) in total and cytosolic cell extracts was measured with the zFR-AFC probe (Enzyme System Products) essentially as described previously [7]. Values were normalized to LDH contents.

### Immunocytochemistry

Immunocytochemistry was performed as described previously [7] using F-actin-binding Alexa Fluor 594-phalloidin, antibodies against α-tubulin (Molecular Probes) or lysosomal-associated membrane protein 2 (Lamp-2; SouthernBiotech) and Alexa Fluor 488/594-coupled secondary antibodies (Molecular Probes).

### Western blot

SDS-PAGE-separated proteins were transferred to membranes that were blocked and incubated with antibodies against Bcl-2 (Abcam), β-actin (Sigma-Aldrich), p62/SQSTM1 (Enzo Life Science), myosin heavy chain 1 (MYH1; Sigma-Aldrich), glyceraldehyde-3-phosphate dehydrogenase (GAPDH; Biogenesis) or heat shock 70 kDa protein 8 (Hsc70; N69, kindly provided by Boris Margulis, Russian Academy of Sciences, St. Petersburg, Russia) and horseradish peroxidase-conjugated secondary antibodies (Dako or Vector Laboratories). Bound antibodies were detected by enhanced chemiluminescence reagents (GE Healthcare) and a LAS-1000Plus luminescent image analyzer (Fujifilm). Band intensities were quantified with ImageJ (http://imagej.nih.gov/ij/).

### TfLC3 puncta formation assay

Autophagic flux was analyzed using MCF7 cells stably expressing a tandem fluorescent construct consisting of LC3 fused with mRFP and eGFP [21] (construct kindly provided by Tamotsu Yoshimori, Osaka University, Japan) as described previously [22].

### Lysosomal stability assay

Lysosomal stability was measured by exposing acridine orange-stained cells to 489 nm laser light and capturing images in green and red channels every 330 ms as described previously [23].

### Statistical analysis

Statistical significance was calculated using two-tailed, paired Student's T-tests.

## Supporting Information

Table S1 **Overview of Silencer® Molecular Motor Library.**
(DOC)

Figure S1 **Validation of the identified cell death-inducing siRNAs by RT-PCR and Western blot.**
(TIF)

Figure S2 **The KIF11 inhibitor monastrol sensitizes cancer cells to siramesine.**
(TIF)
