# Supplementary material for: Identification of Cytoskeleton-Associated Proteins Essential for Lysosomal Stability and Survival of Human Cancer Cells
Source: PLoS One. 2012 Oct 11;7(10):e45381. doi: 10.1371/journal.pone.0045381 (PMC3469574; doi:10.1371/journal.pone.0045381)
Supplement: Table S1 — Overview of Silencer® Molecular Motor Library. (DOC) [file pone.0045381.s001.doc]

Supplementary Table S1. Overview of Silencer® Molecular Motor Library.

| **Gene Symbol** | **Full Gene Name** | **RefSeq Accession Number** | **Sense siRNA Sequence** |
| --- | --- | --- | --- |
| [CENPE](http://www.ncbi.nlm.nih.gov/entrez/query.fcgi?db=gene&cmd=search&term=CENPE) | centromere protein E, 312kDa | NM_001813 | GGAAUUAAAGGCUAAAAGAtt |
| [CENPE](http://www.ncbi.nlm.nih.gov/entrez/query.fcgi?db=gene&cmd=search&term=CENPE) | centromere protein E, 312kDa | NM_001813 | GGAUCUGUUAAGGUAUCCCtt |
| [CENPE](http://www.ncbi.nlm.nih.gov/entrez/query.fcgi?db=gene&cmd=search&term=CENPE) | centromere protein E, 312kDa | NM_001813 | GGAAAAACAUAUACCAUGAtt |
| [CGNL1](http://www.ncbi.nlm.nih.gov/entrez/query.fcgi?db=gene&cmd=search&term=FLJ14957) | cingulin-like 1 | NM_032866 | GGUUCAUAGACUUAGGUGUtt |
| [CGNL1](http://www.ncbi.nlm.nih.gov/entrez/query.fcgi?db=gene&cmd=search&term=FLJ14957) | cingulin-like 1 | NM_032866 | GCCUAGCCCAAUAAGAAACtt |
| [CGNL1](http://www.ncbi.nlm.nih.gov/entrez/query.fcgi?db=gene&cmd=search&term=FLJ14957) | cingulin-like 1 | NM_032866 | GGUCUGGGAAGCGAAACAGtt |
| [custom](http://www.ncbi.nlm.nih.gov/entrez/query.fcgi?db=gene&cmd=search&term=custom) | GRCh37 | NT_010783 | GCAUCUGCUCCCUCAUAAAtt |
| [custom](http://www.ncbi.nlm.nih.gov/entrez/query.fcgi?db=gene&cmd=search&term=custom) | GRCh37 | NT_010783 | CCGUGGUGUCCAAGUUCAAtt |
| [custom](http://www.ncbi.nlm.nih.gov/entrez/query.fcgi?db=gene&cmd=search&term=custom) | GRCh37 | NT_010783 | GACCUUUCUCCAAGAGGAGtt |
| [DCTN2](http://www.ncbi.nlm.nih.gov/entrez/query.fcgi?db=gene&cmd=search&term=DCTN2) | dynactin 2 (p50) | NM_006400 | GGACAGGAUAUGAAUCUGGtt |
| [DCTN2](http://www.ncbi.nlm.nih.gov/entrez/query.fcgi?db=gene&cmd=search&term=DCTN2) | dynactin 2 (p50) | NM_006400 | GGUGCACCAGCUAUAUGAAtt |
| [DCTN2](http://www.ncbi.nlm.nih.gov/entrez/query.fcgi?db=gene&cmd=search&term=DCTN2) | dynactin 2 (p50) | NM_006400 | GGAUGAUCAAGCGGAGUUCtt |
| [DCTN5](http://www.ncbi.nlm.nih.gov/entrez/query.fcgi?db=gene&cmd=search&term=MGC3248) | dynactin 5 (p25) | NM_032486 | GCUAUCCUGACUUAAUAUCtt |
| [DCTN5](http://www.ncbi.nlm.nih.gov/entrez/query.fcgi?db=gene&cmd=search&term=MGC3248) | dynactin 5 (p25) | NM_032486 | GGGCUCUUAGAAUCAGUUUtt |
| [DCTN5](http://www.ncbi.nlm.nih.gov/entrez/query.fcgi?db=gene&cmd=search&term=MGC3248) | dynactin 5 (p25) | NM_032486 | GCUAAUUCGCAUAUAUACAtt |
| [DCTN6](http://www.ncbi.nlm.nih.gov/entrez/query.fcgi?db=gene&cmd=search&term=DCTN6) | dynactin 6 | NM_006571 | GGCUACCCUUGACAAGAAAtt |
| [DCTN6](http://www.ncbi.nlm.nih.gov/entrez/query.fcgi?db=gene&cmd=search&term=DCTN6) | dynactin 6 | NM_006571 | CCACCUAAAGAAGACUAUGtt |
| [DCTN6](http://www.ncbi.nlm.nih.gov/entrez/query.fcgi?db=gene&cmd=search&term=DCTN6) | dynactin 6 | NM_006571 | GGAGAUGUAACUAUCGGACtt |
| [DNAH1](http://www.ncbi.nlm.nih.gov/entrez/query.fcgi?db=gene&cmd=search&term=XLHSRF-1) | dynein, axonemal, heavy chain 1 | NM_015512 | GGUCUUCCAAGGCAUGCUCtt |
| DNAH1 | dynein, axonemal, heavy chain 1 | NM_015512 | GGUCAGCAAGAAACGCAUCtt |
| DNAH1 | dynein, axonemal, heavy chain 1 | NM_015512 | GGAAUCAAUGAACACAGUAtt |
| [DNAH11](http://www.ncbi.nlm.nih.gov/entrez/query.fcgi?db=gene&cmd=search&term=DNAH11) | dynein, axonemal, heavy polypeptide 11 | NM_003777 | CCAUAAGUCCUGGUCCUGUtt |
| [DNAH11](http://www.ncbi.nlm.nih.gov/entrez/query.fcgi?db=gene&cmd=search&term=DNAH11) | dynein, axonemal, heavy polypeptide 11 | NM_003777 | GCAACUGUAUAAUGAACACtt |
| [DNAH11](http://www.ncbi.nlm.nih.gov/entrez/query.fcgi?db=gene&cmd=search&term=DNAH11) | dynein, axonemal, heavy polypeptide 11 | NM_003777 | GCUCGGGUUAUAGUUUUAUtt |
| [DNAH12](http://www.ncbi.nlm.nih.gov/entrez/query.fcgi?db=gene&cmd=search&term=FLJ40427) | dynein, axonemal, heavy chain 12 | NM_178504 | CAAGCUAUCCAUCUUCAAAtt |
| [DNAH12](http://www.ncbi.nlm.nih.gov/entrez/query.fcgi?db=gene&cmd=search&term=FLJ40427) | dynein, axonemal, heavy chain 12 | NM_178504 | GUUACAUCACAGAUUUCCUtt |
| [DNAH12](http://www.ncbi.nlm.nih.gov/entrez/query.fcgi?db=gene&cmd=search&term=FLJ40427) | dynein, axonemal, heavy chain 12 | NM_178504 | CCAAAGAUAUCCUCAACAAtt |
| [DNAH17](http://www.ncbi.nlm.nih.gov/entrez/query.fcgi?db=gene&cmd=search&term=DNAH17) | dynein, axonemal, heavy chain 17 | NM_003727 | CGUGGUGUUUGAGAAAGCCtt |
| [DNAH17](http://www.ncbi.nlm.nih.gov/entrez/query.fcgi?db=gene&cmd=search&term=DNAH17) | dynein, axonemal, heavy chain 17 | NM_003727 | CGUCAAUCUUCUUCAUCCUtt |
| [DNAH17](http://www.ncbi.nlm.nih.gov/entrez/query.fcgi?db=gene&cmd=search&term=DNAH17) | dynein, axonemal, heavy chain 17 | NM_003727 | CCUGAGCAACCUAACCUCAtt |
| [DNAH2](http://www.ncbi.nlm.nih.gov/entrez/query.fcgi?db=gene&cmd=search&term=custom) | dynein, axonemal, heavy chain 2 | NM_020877 | AUCCAUCCUCACCAUCUUCtt |
| DNAH2 | dynein, axonemal, heavy chain 2 | NM_020877 | GGCCAUGAACAUGAAGCCUtt |
| DNAH2 | dynein, axonemal, heavy chain 2 | NM_020877 | GACAGGAGAAAAUUUAGGUtt |
| [DNAH3](http://www.ncbi.nlm.nih.gov/entrez/query.fcgi?db=gene&cmd=search&term=DNAH3) | dynein, axonemal, heavy polypeptide 3 | NM_017539 | CCGUCUGGACUCUAUCAGAtt |
| [DNAH3](http://www.ncbi.nlm.nih.gov/entrez/query.fcgi?db=gene&cmd=search&term=DNAH3) | dynein, axonemal, heavy polypeptide 3 | NM_017539 | GCUGAAAUACAUACCACUUtt |
| [DNAH3](http://www.ncbi.nlm.nih.gov/entrez/query.fcgi?db=gene&cmd=search&term=DNAH3) | dynein, axonemal, heavy polypeptide 3 | NM_017539 | GCUUUUACCCGCCCUUGAUtt |
| [DNAH5](http://www.ncbi.nlm.nih.gov/entrez/query.fcgi?db=gene&cmd=search&term=DNAH5) | dynein, axonemal, heavy polypeptide 5 | NM_001369 | GGGAAUCAGAUUGAAAGAAtt |
| [DNAH5](http://www.ncbi.nlm.nih.gov/entrez/query.fcgi?db=gene&cmd=search&term=DNAH5) | dynein, axonemal, heavy polypeptide 5 | NM_001369 | GCAAAGGACAAUGUGAAAUtt |
| [DNAH5](http://www.ncbi.nlm.nih.gov/entrez/query.fcgi?db=gene&cmd=search&term=DNAH5) | dynein, axonemal, heavy polypeptide 5 | NM_001369 | GGACAACUUGGCUCUCUAGtt |
| [DNAH6](http://www.ncbi.nlm.nih.gov/entrez/query.fcgi?db=gene&cmd=search&term=custom) | dynein, axonemal, heavy chain 6 | NM_001370 | GAGAAGCACUUCUUUCUGUtt |
| DNAH6 | dynein, axonemal, heavy chain 6 | NM_001370 | AAAGCUUCCCUUGAUGUGCtt |
| DNAH6 | dynein, axonemal, heavy chain 6 | NM_001370 | CCUGGAGCUUAUCAAUCUUtt |
| [DNAH7](http://www.ncbi.nlm.nih.gov/entrez/query.fcgi?db=gene&cmd=search&term=DNAH7) | dynein, axonemal, heavy polypeptide 7 | NM_018897 | GCAGUGAGCAGGAUAAAUCtt |
| [DNAH7](http://www.ncbi.nlm.nih.gov/entrez/query.fcgi?db=gene&cmd=search&term=DNAH7) | dynein, axonemal, heavy polypeptide 7 | NM_018897 | GCUUUACCACAGCUGUCUAtt |
| [DNAH7](http://www.ncbi.nlm.nih.gov/entrez/query.fcgi?db=gene&cmd=search&term=DNAH7) | dynein, axonemal, heavy polypeptide 7 | NM_018897 | GCACAAUUCCAAAACCGACtt |
| [DNAH8](http://www.ncbi.nlm.nih.gov/entrez/query.fcgi?db=gene&cmd=search&term=DNAH8) | dynein, axonemal, heavy polypeptide 8 | NM_001371 | GGAAGUGCUGAUGGUAUGGtt |
| [DNAH8](http://www.ncbi.nlm.nih.gov/entrez/query.fcgi?db=gene&cmd=search&term=DNAH8) | dynein, axonemal, heavy polypeptide 8 | NM_001371 | GGUCCACUCACUGAAUUGGtt |
| [DNAH8](http://www.ncbi.nlm.nih.gov/entrez/query.fcgi?db=gene&cmd=search&term=DNAH8) | dynein, axonemal, heavy polypeptide 8 | NM_001371 | CCUUGAGUAAUUCUACCAUtt |
| [DNAH9](http://www.ncbi.nlm.nih.gov/entrez/query.fcgi?db=gene&cmd=search&term=DNAH9) | dynein, axonemal, heavy polypeptide 9 | NM_004662 | GGCCUUCAGUAUCGUCUUCtt |
| [DNAH9](http://www.ncbi.nlm.nih.gov/entrez/query.fcgi?db=gene&cmd=search&term=DNAH9) | dynein, axonemal, heavy polypeptide 9 | NM_004662 | GCUGUCAAGGUACUUUCAUtt |
| [DNAH9](http://www.ncbi.nlm.nih.gov/entrez/query.fcgi?db=gene&cmd=search&term=DNAH9) | dynein, axonemal, heavy polypeptide 9 | NM_001372 | CGACCUCAGCAAGAUCCAUtt |
| [DNAI1](http://www.ncbi.nlm.nih.gov/entrez/query.fcgi?db=gene&cmd=search&term=DNAI1) | dynein, axonemal, intermediate polypeptide 1 | NM_012144 | GGAGGAGUUCACUCGGAUUtt |
| [DNAI1](http://www.ncbi.nlm.nih.gov/entrez/query.fcgi?db=gene&cmd=search&term=DNAI1) | dynein, axonemal, intermediate polypeptide 1 | NM_012144 | GCUUCCCUGAGUACAUGUUtt |
| [DNAI1](http://www.ncbi.nlm.nih.gov/entrez/query.fcgi?db=gene&cmd=search&term=DNAI1) | dynein, axonemal, intermediate polypeptide 1 | NM_012144 | CCGAUGCGGAGUUAAAGGAtt |
| [DNAI2](http://www.ncbi.nlm.nih.gov/entrez/query.fcgi?db=gene&cmd=search&term=DNAI2) | dynein, axonemal, intermediate chain 2 | NM_023036 | GCGGUUUGAGAUGAAGACCtt |
| [DNAI2](http://www.ncbi.nlm.nih.gov/entrez/query.fcgi?db=gene&cmd=search&term=DNAI2) | dynein, axonemal, intermediate chain 2 | NM_023036 | GCACUGCAUCAAGCAGAACtt |
| [DNAI2](http://www.ncbi.nlm.nih.gov/entrez/query.fcgi?db=gene&cmd=search&term=DNAI2) | dynein, axonemal, intermediate chain 2 | NM_023036 | CGUAGCCUCUUCCAUGUUUtt |
| [DNAL4](http://www.ncbi.nlm.nih.gov/entrez/query.fcgi?db=gene&cmd=search&term=DNAL4) | dynein, axonemal, light polypeptide 4 | NM_005740 | GGGUUUUAGUAGAAGGGUGtt |
| [DNAL4](http://www.ncbi.nlm.nih.gov/entrez/query.fcgi?db=gene&cmd=search&term=DNAL4) | dynein, axonemal, light polypeptide 4 | NM_005740 | CCAUGGAGCUAUGUGUCACtt |
| [DNAL4](http://www.ncbi.nlm.nih.gov/entrez/query.fcgi?db=gene&cmd=search&term=DNAL4) | dynein, axonemal, light polypeptide 4 | NM_005740 | GCUAUGUGUCACAGCCUGUtt |
| [DNCI1](http://www.ncbi.nlm.nih.gov/entrez/query.fcgi?db=gene&cmd=search&term=DNCI1) | dynein, cytoplasmic, intermediate polypeptide 1 | NM_004411 | GGAAGAGGAGAGGAAAAAGtt |
| [DNCI1](http://www.ncbi.nlm.nih.gov/entrez/query.fcgi?db=gene&cmd=search&term=DNCI1) | dynein, cytoplasmic, intermediate polypeptide 1 | NM_004411 | GGUUAUAUUAAGUUCCACAtt |
| [DNCI1](http://www.ncbi.nlm.nih.gov/entrez/query.fcgi?db=gene&cmd=search&term=DNCI1) | dynein, cytoplasmic, intermediate polypeptide 1 | NM_004411 | CCACUAAUACUUCUCACUGtt |
| [DNCI2](http://www.ncbi.nlm.nih.gov/entrez/query.fcgi?db=gene&cmd=search&term=DNCI2) | dynein, cytoplasmic, intermediate polypepide 2 | NM_001378 | CCAUUCUACAAGAAUUGUAtt |
| [DNCI2](http://www.ncbi.nlm.nih.gov/entrez/query.fcgi?db=gene&cmd=search&term=DNCI2) | dynein, cytoplasmic, intermediate polypepide 2 | NM_001378 | CGAGGACCUAUUAAACUUGtt |
| [DNCI2](http://www.ncbi.nlm.nih.gov/entrez/query.fcgi?db=gene&cmd=search&term=DNCI2) | dynein, cytoplasmic, intermediate polypepide 2 | NM_001378 | GGACCUAUUAAACUUGGAAtt |
| [DNCL2B](http://www.ncbi.nlm.nih.gov/entrez/query.fcgi?db=gene&cmd=search&term=DNCL2B) | dynein, cytoplasmic, light polypeptide 2B | NM_130897 | GGCCUUCUUCAUCACCUGAtt |
| [DNCL2B](http://www.ncbi.nlm.nih.gov/entrez/query.fcgi?db=gene&cmd=search&term=DNCL2B) | dynein, cytoplasmic, light polypeptide 2B | NM_130897 | GGCUGUUUAAGCGACACUGtt |
| [DNCL2B](http://www.ncbi.nlm.nih.gov/entrez/query.fcgi?db=gene&cmd=search&term=DNCL2B) | dynein, cytoplasmic, light polypeptide 2B | NM_130897 | GGUAUUCCCAUCCGAACAAtt |
| [DNCLI1](http://www.ncbi.nlm.nih.gov/entrez/query.fcgi?db=gene&cmd=search&term=DNCLI1) | dynein, cytoplasmic, light intermediate chain 1 | NM_016141 | GGAAGAGGAUUGGAAUAUUtt |
| [DNCLI1](http://www.ncbi.nlm.nih.gov/entrez/query.fcgi?db=gene&cmd=search&term=DNCLI1) | dynein, cytoplasmic, light intermediate chain 1 | NM_016141 | GCCGUACAGAAGAAAAAUUtt |
| [DNCLI1](http://www.ncbi.nlm.nih.gov/entrez/query.fcgi?db=gene&cmd=search&term=DNCLI1) | dynein, cytoplasmic, light intermediate chain 1 | NM_016141 | GGGAAUAGAGGAGUAUAAGtt |
| [DNCLI2](http://www.ncbi.nlm.nih.gov/entrez/query.fcgi?db=gene&cmd=search&term=DNCLI2) | dynein, cytoplasmic, light intermediate chain 2 | NM_006141 | GCCGGACCCAAACAUCAAAtt |
| [DNCLI2](http://www.ncbi.nlm.nih.gov/entrez/query.fcgi?db=gene&cmd=search&term=DNCLI2) | dynein, cytoplasmic, light intermediate chain 2 | NM_006141 | CGGAAGUUUGUGAAAGAUUtt |
| [DNCLI2](http://www.ncbi.nlm.nih.gov/entrez/query.fcgi?db=gene&cmd=search&term=DNCLI2) | dynein, cytoplasmic, light intermediate chain 2 | NM_006141 | GGAGCUGAGCAUGGCAAAAtt |
| [DYNC1H1](http://www.ncbi.nlm.nih.gov/entrez/query.fcgi?db=gene&cmd=search&term=DNCH1) | dynein, cytoplasmic, heavy polypeptide 1 | NM_001376 | GCAAAAUAUUGAAAUUCCGtt |
| [DYNC1H1](http://www.ncbi.nlm.nih.gov/entrez/query.fcgi?db=gene&cmd=search&term=DNCH1) | dynein, cytoplasmic, heavy polypeptide 1 | NM_001376 | CGUACUCCCGUGAUUGAUGtt |
| [DYNC1H1](http://www.ncbi.nlm.nih.gov/entrez/query.fcgi?db=gene&cmd=search&term=DNCH1) | dynein, cytoplasmic, heavy polypeptide 1 | NM_001376 | GCAGUGGAUGACUUAAAUCtt |
| [DYNC2H1](http://www.ncbi.nlm.nih.gov/entrez/query.fcgi?db=gene&cmd=search&term=FLJ11756) | dynein, cytoplasmic, heavy polypeptide 2 | XM_370652 | CCACUUUCUGGCAAAAAUCtt |
| DYNC2H1 | dynein, cytoplasmic, heavy polypeptide 2 | NM_024606 | GGCUGAUCAGUUGAUGUUCtt |
| DYNC2H1 | dynein, cytoplasmic, heavy polypeptide 2 | XM_370652 | GCUGGUAUUUCAAUUUGUGtt |
| [DYNLL1](http://www.ncbi.nlm.nih.gov/entrez/query.fcgi?db=gene&cmd=search&term=custom) | dynein, light chain, LC8-type 1 | NM_003746 | UAUCAAGAAGGAAUUUGACtt |
| DYNLL1 | dynein, light chain, LC8-type 1 | NM_003746 | UUCUUCUGUUCAAAUCUGGtt |
| DYNLL1 | dynein, light chain, LC8-type 1 | NM_003746 | GUGAUCCAUCCAAAAACAAtt |
| [DYNLL2](http://www.ncbi.nlm.nih.gov/entrez/query.fcgi?db=gene&cmd=search&term=Dlc2) | dynein, light chain, LC8-type 2 | NM_080677 | CGAGACAAAGCACUUCAUCtt |
| DYNLL2 | dynein, light chain, LC8-type 2 | NM_080677 | CCCUACCUGGCAUUGUAUCtt |
| DYNLL2 | dynein, light chain, LC8-type 2 | NM_080677 | GGCCAUGGAGAAGUACAAUtt |
| [DYNLRB1](http://www.ncbi.nlm.nih.gov/entrez/query.fcgi?db=gene&cmd=search&term=DNCL2A) | dynein, light chain, roadblock-type 1 | NM_014183 | CCUUCAGUUCACUUUGUCGtt |
| DYNLRB1 | dynein, light chain, roadblock-type 1 | NM_014183 | GAAUGUUAAUGUCAAUCAUtt |
| DYNLRB1 | dynein, light chain, roadblock-type 1 | NM_014183 | GGUUUUGGAGCAAGAGCUUtt |
| [DYNLT1](http://www.ncbi.nlm.nih.gov/entrez/query.fcgi?db=gene&cmd=search&term=TCTEL1) | dynein, light chain, Tctex-type 1 | NM_006519 | CCAAAUGACCGCACUGUGAtt |
| DYNLT1 | dynein, light chain, Tctex-type 1 | NM_006519 | GGCUAUAGAAAGCGCAAUUtt |
| DYNLT1 | dynein, light chain, Tctex-type 1 | NM_006519 | CCAUGUACUGCAUCGUCAGtt |
| [DYNLT3](http://www.ncbi.nlm.nih.gov/entrez/query.fcgi?db=gene&cmd=search&term=TCTE1L) | dynein, light chain, Tctex-type 3 | NM_006520 | GCAUCGUUUAAAUAGGAAAtt |
| DYNLT3 | dynein, light chain, Tctex-type 3 | NM_006520 | GCGUGCAAUAUGCUUUAAAtt |
| DYNLT3 | dynein, light chain, Tctex-type 3 | NM_006520 | GGGAGGAAAAAAGUGAAUCtt |
| [KIF11](http://www.ncbi.nlm.nih.gov/entrez/query.fcgi?db=gene&cmd=search&term=KIF11) | kinesin family member 11 | NM_004523 | GCUACUCUGAUGAAUGCAUtt |
| [KIF11](http://www.ncbi.nlm.nih.gov/entrez/query.fcgi?db=gene&cmd=search&term=KIF11) | kinesin family member 11 | NM_004523 | GGUGAAAGGUCACCUAAUGtt |
| [KIF11](http://www.ncbi.nlm.nih.gov/entrez/query.fcgi?db=gene&cmd=search&term=KIF11) | kinesin family member 11 | NM_004523 | CUGAAGACCUGAAGACAAUtt |
| [KIF13A](http://www.ncbi.nlm.nih.gov/entrez/query.fcgi?db=gene&cmd=search&term=KIF13A) | kinesin family member 13A | NM_022113 | CCAUAUGUAGAUGGUUUAUtt |
| [KIF13A](http://www.ncbi.nlm.nih.gov/entrez/query.fcgi?db=gene&cmd=search&term=KIF13A) | kinesin family member 13A | NM_022113 | GGGAAUCAAACGGUCCUGCtt |
| [KIF13A](http://www.ncbi.nlm.nih.gov/entrez/query.fcgi?db=gene&cmd=search&term=KIF13A) | kinesin family member 13A | NM_022113 | GGAAACCUCCCAAGGUAUUtt |
| [KIF13B](http://www.ncbi.nlm.nih.gov/entrez/query.fcgi?db=gene&cmd=search&term=KIF13B) | kinesin family member 13B | NM_015254 | GGCUUAUAUUGAAAAGUACtt |
| [KIF13B](http://www.ncbi.nlm.nih.gov/entrez/query.fcgi?db=gene&cmd=search&term=KIF13B) | kinesin family member 13B | NM_015254 | GCUAAUCCAGGAAAUGACUtt |
| [KIF13B](http://www.ncbi.nlm.nih.gov/entrez/query.fcgi?db=gene&cmd=search&term=KIF13B) | kinesin family member 13B | NM_015254 | GGUCAAGAUAUUGUUUUCAtt |
| [KIF14](http://www.ncbi.nlm.nih.gov/entrez/query.fcgi?db=gene&cmd=search&term=KIF14) | kinesin family member 14 | NM_014875 | GGUGAAACAGAAAAUAAUGtt |
| [KIF14](http://www.ncbi.nlm.nih.gov/entrez/query.fcgi?db=gene&cmd=search&term=KIF14) | kinesin family member 14 | NM_014875 | GGUAAAGUCAGAGACAUAAtt |
| [KIF14](http://www.ncbi.nlm.nih.gov/entrez/query.fcgi?db=gene&cmd=search&term=KIF14) | kinesin family member 14 | NM_014875 | GGAGAACUACAAGGAACAAtt |
| [KIF17](http://www.ncbi.nlm.nih.gov/entrez/query.fcgi?db=gene&cmd=search&term=KIF17) | kinesin family member 17 | NM_020816 | CCCUCCAAAUCUGAGAUUUtt |
| [KIF17](http://www.ncbi.nlm.nih.gov/entrez/query.fcgi?db=gene&cmd=search&term=KIF17) | kinesin family member 17 | NM_020816 | GCAGAUCUACAACGAGAUCtt |
| [KIF17](http://www.ncbi.nlm.nih.gov/entrez/query.fcgi?db=gene&cmd=search&term=KIF17) | kinesin family member 17 | NM_020816 | CCGAGCAGAUCUACAACGAtt |
| [KIF18A](http://www.ncbi.nlm.nih.gov/entrez/query.fcgi?db=gene&cmd=search&term=KIF18A) | kinesin family member 18A | NM_031217 | GCUGGAUUUCAUAAAGUGGtt |
| [KIF18A](http://www.ncbi.nlm.nih.gov/entrez/query.fcgi?db=gene&cmd=search&term=KIF18A) | kinesin family member 18A | NM_031217 | GCCAAUUCUUCGUAGUUUUtt |
| [KIF18A](http://www.ncbi.nlm.nih.gov/entrez/query.fcgi?db=gene&cmd=search&term=KIF18A) | kinesin family member 18A | NM_031217 | GCAGCUGGAUUUCAUAAAGtt |
| [KIF19](http://www.ncbi.nlm.nih.gov/entrez/query.fcgi?db=gene&cmd=search&term=FLJ37300) | kinesin family member 19 | NM_153209 | GCGACUCAAGCGCAAGAUUtt |
| [KIF19](http://www.ncbi.nlm.nih.gov/entrez/query.fcgi?db=gene&cmd=search&term=FLJ37300) | kinesin family member 19 | NM_153209 | GGUGAUGACCAACCAGACAtt |
| [KIF19](http://www.ncbi.nlm.nih.gov/entrez/query.fcgi?db=gene&cmd=search&term=FLJ37300) | kinesin family member 19 | NM_153209 | GGCUGUGGGAAAACCUACAtt |
| [KIF1A](http://www.ncbi.nlm.nih.gov/entrez/query.fcgi?db=gene&cmd=search&term=KIF1A) | kinesin family member 1A | NM_004321 | GGAAACAGAGAAGAUCAUAtt |
| [KIF1A](http://www.ncbi.nlm.nih.gov/entrez/query.fcgi?db=gene&cmd=search&term=KIF1A) | kinesin family member 1A | NM_004321 | CCAACGACAACAUGUCCUAtt |
| [KIF1A](http://www.ncbi.nlm.nih.gov/entrez/query.fcgi?db=gene&cmd=search&term=KIF1A) | kinesin family member 1A | NM_004321 | GGUCAGCUACAUGGAGAUUtt |
| [KIF1B](http://www.ncbi.nlm.nih.gov/entrez/query.fcgi?db=gene&cmd=search&term=KIF1B) | kinesin family member 1B | NM_183416 | GGAAUCCAAAUGCAUCAUUtt |
| [KIF1B](http://www.ncbi.nlm.nih.gov/entrez/query.fcgi?db=gene&cmd=search&term=KIF1B) | kinesin family member 1B | NM_015074 | GGGAUAUAAUGUCUGUAUUtt |
| [KIF1B](http://www.ncbi.nlm.nih.gov/entrez/query.fcgi?db=gene&cmd=search&term=KIF1B) | kinesin family member 1B | NM_183416 | GGAAGCUCCAAAGUCCUUCtt |
| [KIF1C](http://www.ncbi.nlm.nih.gov/entrez/query.fcgi?db=gene&cmd=search&term=KIF1C) | kinesin family member 1C | NM_006612 | GGAUAGCAAACAGGAAAAAtt |
| [KIF1C](http://www.ncbi.nlm.nih.gov/entrez/query.fcgi?db=gene&cmd=search&term=KIF1C) | kinesin family member 1C | NM_006612 | GGUGAGCUAUAUGGAGAUCtt |
| [KIF1C](http://www.ncbi.nlm.nih.gov/entrez/query.fcgi?db=gene&cmd=search&term=KIF1C) | kinesin family member 1C | NM_006612 | GCGAAAGUCGGAUUUUAUCtt |
| [KIF2](http://www.ncbi.nlm.nih.gov/entrez/query.fcgi?db=gene&cmd=search&term=KIF2) | kinesin heavy chain member 2 | NM_004520 | GCUGUGCUCACUUUUCCUGtt |
| [KIF2](http://www.ncbi.nlm.nih.gov/entrez/query.fcgi?db=gene&cmd=search&term=KIF2) | kinesin heavy chain member 2 | NM_004520 | GGCCCUCUUAGAGAUGACUtt |
| [KIF2](http://www.ncbi.nlm.nih.gov/entrez/query.fcgi?db=gene&cmd=search&term=KIF2) | kinesin heavy chain member 2 | NM_004520 | GCCAAAGUAAACAAAAUUGtt |
| [KIF20A](http://www.ncbi.nlm.nih.gov/entrez/query.fcgi?db=gene&cmd=search&term=KIF20A) | kinesin family member 20A | NM_005733 | CCUGCUAUCAGACUGCUCUtt |
| [KIF20A](http://www.ncbi.nlm.nih.gov/entrez/query.fcgi?db=gene&cmd=search&term=KIF20A) | kinesin family member 20A | NM_005733 | GCAGCAGGUUCCAUCUGAGtt |
| [KIF20A](http://www.ncbi.nlm.nih.gov/entrez/query.fcgi?db=gene&cmd=search&term=KIF20A) | kinesin family member 20A | NM_005733 | GGUUAAAGCUAAAUUACAGtt |
| [KIF20B](http://www.ncbi.nlm.nih.gov/entrez/query.fcgi?db=gene&cmd=search&term=MPHOSPH1) | kinesin family member 20B | NM_016195 | GGGCUGUGUGCAUAUUCUGtt |
| KIF20B | kinesin family member 20B | NM_016195 | GCUUGAUCUGUCUCAUGAAtt |
| KIF20B | kinesin family member 20B | NM_016195 | GGGACAGAAGAAAAUAUUGtt |
| [KIF21A](http://www.ncbi.nlm.nih.gov/entrez/query.fcgi?db=gene&cmd=search&term=KIF21A) | kinesin family member 21A | NM_017641 | GGAUACAAUGCUACAGUUUtt |
| [KIF21A](http://www.ncbi.nlm.nih.gov/entrez/query.fcgi?db=gene&cmd=search&term=KIF21A) | kinesin family member 21A | NM_017641 | GGUCUUCCUAGGGAAAGAUtt |
| [KIF21A](http://www.ncbi.nlm.nih.gov/entrez/query.fcgi?db=gene&cmd=search&term=KIF21A) | kinesin family member 21A | NM_017641 | GGAUGCCAUAUUUGUACAUtt |
| [KIF22](http://www.ncbi.nlm.nih.gov/entrez/query.fcgi?db=gene&cmd=search&term=KIF22) | kinesin family member 22 | NM_007317 | GCUGCUCUCUAGAGAUUGCtt |
| [KIF22](http://www.ncbi.nlm.nih.gov/entrez/query.fcgi?db=gene&cmd=search&term=KIF22) | kinesin family member 22 | NM_007317 | GCUGUCUCAGAAAGAAUUGtt |
| [KIF22](http://www.ncbi.nlm.nih.gov/entrez/query.fcgi?db=gene&cmd=search&term=KIF22) | kinesin family member 22 | NM_007317 | GCAAGAUUGGAGCUACUCGtt |
| [KIF23](http://www.ncbi.nlm.nih.gov/entrez/query.fcgi?db=gene&cmd=search&term=KIF23) | kinesin family member 23 | NM_138555 | GGUUGAUGCCUUAUUAGAAtt |
| [KIF23](http://www.ncbi.nlm.nih.gov/entrez/query.fcgi?db=gene&cmd=search&term=KIF23) | kinesin family member 23 | NM_004856 | GGAGACUCAGUAUUCAUUUtt |
| [KIF23](http://www.ncbi.nlm.nih.gov/entrez/query.fcgi?db=gene&cmd=search&term=KIF23) | kinesin family member 23 | NM_004856 | GGAACUCUUUGAUGUUGUGtt |
| [KIF24](http://www.ncbi.nlm.nih.gov/entrez/query.fcgi?db=gene&cmd=search&term=custom) | kinesin family member 24 | AK001795 | GACAGCUGAAUUUUGAUUCtt |
| KIF24 | kinesin family member 24 | AK001795 | CAAUAAAGACAGAAAUGCCtt |
| KIF24 | kinesin family member 24 | AK001795 | CAGCAUCUACUCCUUAUUUtt |
| [KIF25](http://www.ncbi.nlm.nih.gov/entrez/query.fcgi?db=gene&cmd=search&term=KIF25) | kinesin family member 25 | NM_030615 | GGUUGAAGUCUCCAUAGUGtt |
| [KIF25](http://www.ncbi.nlm.nih.gov/entrez/query.fcgi?db=gene&cmd=search&term=KIF25) | kinesin family member 25 | NM_030615 | GGCUCAUUUUGGAAAAUACtt |
| [KIF25](http://www.ncbi.nlm.nih.gov/entrez/query.fcgi?db=gene&cmd=search&term=KIF25) | kinesin family member 25 | NM_030615 | GCGGAAAGAGCUAUACCAUtt |
| [KIF27](http://www.ncbi.nlm.nih.gov/entrez/query.fcgi?db=gene&cmd=search&term=KIF27) | kinesin family member 27 | NM_017576 | GCAUUUGUCAAGUUCAUAAtt |
| [KIF27](http://www.ncbi.nlm.nih.gov/entrez/query.fcgi?db=gene&cmd=search&term=KIF27) | kinesin family member 27 | NM_017576 | GGAGUGGUUUAACAUGAUCtt |
| [KIF27](http://www.ncbi.nlm.nih.gov/entrez/query.fcgi?db=gene&cmd=search&term=KIF27) | kinesin family member 27 | NM_017576 | GGAUCUUCACAUCCGAGAAtt |
| [KIF2B](http://www.ncbi.nlm.nih.gov/entrez/query.fcgi?db=gene&cmd=search&term=LOC84643) | kinesin family member 2B | NM_032559 | CGGAGAUCAACAGAGAAAAtt |
| [KIF2B](http://www.ncbi.nlm.nih.gov/entrez/query.fcgi?db=gene&cmd=search&term=LOC84643) | kinesin family member 2B | NM_032559 | CCAUGAUAUUGAUUUUUGCtt |
| [KIF2B](http://www.ncbi.nlm.nih.gov/entrez/query.fcgi?db=gene&cmd=search&term=LOC84643) | kinesin family member 2B | NM_032559 | GGCAAGAAGAUUGACCUGGtt |
| [KIF2C](http://www.ncbi.nlm.nih.gov/entrez/query.fcgi?db=gene&cmd=search&term=KIF2C) | kinesin family member 2C | NM_006845 | GGAAGUGGAAAAAAUGAAGtt |
| [KIF2C](http://www.ncbi.nlm.nih.gov/entrez/query.fcgi?db=gene&cmd=search&term=KIF2C) | kinesin family member 2C | NM_006845 | GGGCAAAGAGAUUGAUUUUtt |
| [KIF2C](http://www.ncbi.nlm.nih.gov/entrez/query.fcgi?db=gene&cmd=search&term=KIF2C) | kinesin family member 2C | NM_006845 | GCAACUUGUUUUGCAUAUGtt |
| [KIF3A](http://www.ncbi.nlm.nih.gov/entrez/query.fcgi?db=gene&cmd=search&term=KIF3A) | kinesin family member 3A | NM_007054 | GGUGUUCGAGCUAUUCCUGtt |
| [KIF3A](http://www.ncbi.nlm.nih.gov/entrez/query.fcgi?db=gene&cmd=search&term=KIF3A) | kinesin family member 3A | NM_007054 | CCUCCAAAGACAUUUACUUtt |
| [KIF3A](http://www.ncbi.nlm.nih.gov/entrez/query.fcgi?db=gene&cmd=search&term=KIF3A) | kinesin family member 3A | NM_007054 | GGCUACAAUGGGACUAUUUtt |
| [KIF3B](http://www.ncbi.nlm.nih.gov/entrez/query.fcgi?db=gene&cmd=search&term=KIF3B) | kinesin family member 3B | NM_004798 | CCAAAAAACUCAAAAAGCUtt |
| [KIF3B](http://www.ncbi.nlm.nih.gov/entrez/query.fcgi?db=gene&cmd=search&term=KIF3B) | kinesin family member 3B | NM_004798 | GCAGUUUGAACUGUACGAUtt |
| [KIF3B](http://www.ncbi.nlm.nih.gov/entrez/query.fcgi?db=gene&cmd=search&term=KIF3B) | kinesin family member 3B | NM_004798 | CCUUUGAUGCCGUCUAUGAtt |
| [KIF3C](http://www.ncbi.nlm.nih.gov/entrez/query.fcgi?db=gene&cmd=search&term=KIF3C) | kinesin family member 3C | NM_002254 | GGAAAUCACAAUUUUCAUCtt |
| [KIF3C](http://www.ncbi.nlm.nih.gov/entrez/query.fcgi?db=gene&cmd=search&term=KIF3C) | kinesin family member 3C | NM_002254 | CCCUUCUUACAUCAUUCAAtt |
| [KIF3C](http://www.ncbi.nlm.nih.gov/entrez/query.fcgi?db=gene&cmd=search&term=KIF3C) | kinesin family member 3C | NM_002254 | GCACAUCUUCACCCACAUCtt |
| [KIF4A](http://www.ncbi.nlm.nih.gov/entrez/query.fcgi?db=gene&cmd=search&term=KIF4A) | kinesin family member 4A | NM_012310 | GCAAGCGAAUGAAAAAAUGtt |
| [KIF4A](http://www.ncbi.nlm.nih.gov/entrez/query.fcgi?db=gene&cmd=search&term=KIF4A) | kinesin family member 4A | NM_012310 | GCGAAUGAAAAAAUGAACGtt |
| [KIF4A](http://www.ncbi.nlm.nih.gov/entrez/query.fcgi?db=gene&cmd=search&term=KIF4A) | kinesin family member 4A | NM_012310 | GGUAAUAGCCAUACUCUUAtt |
| [KIF4B](http://www.ncbi.nlm.nih.gov/entrez/query.fcgi?db=gene&cmd=search&term=LOC285643) | kinesin family member 4B | XM_209695 | GCAAGUGAAUGAAAAACUGtt |
| KIF4B | kinesin family member 4B | XM_209695 | CCAACAGUUGGCAUUAUUCtt |
| KIF4B | kinesin family member 4B | XM_209695 | GGUAACAGCCACACUCUUAtt |
| [KIF5A](http://www.ncbi.nlm.nih.gov/entrez/query.fcgi?db=gene&cmd=search&term=KIF5A) | kinesin family member 5A | NM_004984 | GCAAAAUCAAAUCAGAAGUtt |
| [KIF5A](http://www.ncbi.nlm.nih.gov/entrez/query.fcgi?db=gene&cmd=search&term=KIF5A) | kinesin family member 5A | NM_004984 | GGGAAAACACAUACCAUGGtt |
| [KIF5A](http://www.ncbi.nlm.nih.gov/entrez/query.fcgi?db=gene&cmd=search&term=KIF5A) | kinesin family member 5A | NM_004984 | GCAAGUUUAUCAUGCAUGUtt |
| [KIF5B](http://www.ncbi.nlm.nih.gov/entrez/query.fcgi?db=gene&cmd=search&term=KIF5B) | kinesin family member 5B | NM_004521 | GCACAUCUCAAGAGCAAGUtt |
| [KIF5B](http://www.ncbi.nlm.nih.gov/entrez/query.fcgi?db=gene&cmd=search&term=KIF5B) | kinesin family member 5B | NM_004521 | GCUGAGUGGAAAACUUUAUtt |
| [KIF5B](http://www.ncbi.nlm.nih.gov/entrez/query.fcgi?db=gene&cmd=search&term=KIF5B) | kinesin family member 5B | NM_004521 | GCCUUAUGCAUUUGAUCGGtt |
| [KIF5C](http://www.ncbi.nlm.nih.gov/entrez/query.fcgi?db=gene&cmd=search&term=KIF5C) | kinesin family member 5C | XM_377774 | CCUGGAGUUUCACAUAAAGtt |
| [KIF5C](http://www.ncbi.nlm.nih.gov/entrez/query.fcgi?db=gene&cmd=search&term=KIF5C) | kinesin family member 5C | XM_377774 | GCUAAGACCAUCAAGAAUAtt |
| [KIF5C](http://www.ncbi.nlm.nih.gov/entrez/query.fcgi?db=gene&cmd=search&term=KIF5C) | kinesin family member 5C | XM_377774 | CCACCAUCGUCAUUUGCUGtt |
| [KIF6](http://www.ncbi.nlm.nih.gov/entrez/query.fcgi?db=gene&cmd=search&term=C6orf102) | kinesin family member 6 | NM_145027 | CCAUUCAUUUGUCAAGCAAtt |
| KIF6 | kinesin family member 6 | NM_145027 | GGAAGAAAAGAGAAGGUAUtt |
| KIF6 | kinesin family member 6 | NM_145027 | CGUGAUUUGGCAGAUGGGUtt |
| [KIFAP3](http://www.ncbi.nlm.nih.gov/entrez/query.fcgi?db=gene&cmd=search&term=KIFAP3) | kinesin-associated protein 3 | NM_014970 | GCUACCAUUCUUGGAGAAAtt |
| [KIFAP3](http://www.ncbi.nlm.nih.gov/entrez/query.fcgi?db=gene&cmd=search&term=KIFAP3) | kinesin-associated protein 3 | NM_014970 | GCACUCAUUGUUCACUAUGtt |
| [KIFAP3](http://www.ncbi.nlm.nih.gov/entrez/query.fcgi?db=gene&cmd=search&term=KIFAP3) | kinesin-associated protein 3 | NM_014970 | GCUCUGUGUAUGAAUAUUAtt |
| [KIFC1](http://www.ncbi.nlm.nih.gov/entrez/query.fcgi?db=gene&cmd=search&term=KIFC1) | kinesin family member C1 | NM_002263 | CCUAAAUGCAGAACUAAAAtt |
| [KIFC1](http://www.ncbi.nlm.nih.gov/entrez/query.fcgi?db=gene&cmd=search&term=KIFC1) | kinesin family member C1 | NM_002263 | GGCCAGACCACAGCUCAAAtt |
| [KIFC1](http://www.ncbi.nlm.nih.gov/entrez/query.fcgi?db=gene&cmd=search&term=KIFC1) | kinesin family member C1 | NM_002263 | CGACCAAAAUUACCACAUCtt |
| [KIFC2](http://www.ncbi.nlm.nih.gov/entrez/query.fcgi?db=gene&cmd=search&term=KIFC2) | kinesin family member C2 | NM_145754 | CGGACUCAGAGAAAAGGGUtt |
| [KIFC2](http://www.ncbi.nlm.nih.gov/entrez/query.fcgi?db=gene&cmd=search&term=KIFC2) | kinesin family member C2 | NM_145754 | GCAUCUGACUCUGGAGAACtt |
| [KIFC2](http://www.ncbi.nlm.nih.gov/entrez/query.fcgi?db=gene&cmd=search&term=KIFC2) | kinesin family member C2 | NM_145754 | GGGUUCAGCAUCUGACUCUtt |
| [KIFC3](http://www.ncbi.nlm.nih.gov/entrez/query.fcgi?db=gene&cmd=search&term=KIFC3) | kinesin family member C3 | NM_005550 | GGUGCUGAAGGAGAUGGAAtt |
| [KIFC3](http://www.ncbi.nlm.nih.gov/entrez/query.fcgi?db=gene&cmd=search&term=KIFC3) | kinesin family member C3 | NM_005550 | GGAGAUGGAACAGCAGCUGtt |
| [KIFC3](http://www.ncbi.nlm.nih.gov/entrez/query.fcgi?db=gene&cmd=search&term=KIFC3) | kinesin family member C3 | NM_005550 | CCUGUCAAGUAUGUCAUCAtt |
| [KLC1](http://www.ncbi.nlm.nih.gov/entrez/query.fcgi?db=gene&cmd=search&term=KNS2) | kinesin light chain 1 | NM_182923 | GCACAAUUCCAUUUUACAAtt |
| KLC1 | kinesin light chain 1 | NM_182923 | GCAUCUGGAGUUUAUGAAUtt |
| KLC1 | kinesin light chain 1 | NM_005552 | GCUUUGAAGAAUGAGCACAtt |
| [KLC2](http://www.ncbi.nlm.nih.gov/entrez/query.fcgi?db=gene&cmd=search&term=KLC2) | kinesin light chain 2 | NM_022822 | GCACUUGCUGUUCAUGAGCtt |
| [KLC2](http://www.ncbi.nlm.nih.gov/entrez/query.fcgi?db=gene&cmd=search&term=KLC2) | kinesin light chain 2 | NM_022822 | GCUCCAUCCCUUAUUUAUUtt |
| [KLC2](http://www.ncbi.nlm.nih.gov/entrez/query.fcgi?db=gene&cmd=search&term=KLC2) | kinesin light chain 2 | NM_022822 | GCAGCACUUGCUGUUCAUGtt |
| [KLC3](http://www.ncbi.nlm.nih.gov/entrez/query.fcgi?db=gene&cmd=search&term=KLC2L) | kinesin light chain 3 | NM_177417 | GAUGCUGGAGGAAAAGCAGtt |
| KLC3 | kinesin light chain 3 | NM_145275 | GCAGCAGGUGGUGAGCCACtt |
| KLC3 | kinesin light chain 3 | NM_177417 | GGAAAAGCAGCAGGUGGUGtt |
| [KLC4](http://www.ncbi.nlm.nih.gov/entrez/query.fcgi?db=gene&cmd=search&term=custom) | kinesin, light chain 4 | NM_138343 | GUCGGAGAAACAGAAGCUGtt |
| KLC4 | kinesin, light chain 4 | NM_138343 | GACCAUUGAGUGUCUGCAGtt |
| KLC4 | kinesin, light chain 4 | NM_138343 | CCUCUUUCCUAAUGAGGAGtt |
| [KTN1](http://www.ncbi.nlm.nih.gov/entrez/query.fcgi?db=gene&cmd=search&term=KTN1) | kinectin 1 (kinesin receptor) | NM_004986 | GGCUCAACAGUUAUCUAUCtt |
| [KTN1](http://www.ncbi.nlm.nih.gov/entrez/query.fcgi?db=gene&cmd=search&term=KTN1) | kinectin 1 (kinesin receptor) | NM_182926 | GGCUCUUUGUGUUGUAGACtt |
| [KTN1](http://www.ncbi.nlm.nih.gov/entrez/query.fcgi?db=gene&cmd=search&term=KTN1) | kinectin 1 (kinesin receptor) | NM_004986 | GGACAAGUUACUCGCUGCUtt |
| [LOC124685](http://www.ncbi.nlm.nih.gov/entrez/query.fcgi?db=gene&cmd=search&term=LOC124685) | hCG1644301 | XM_064265 | AGGCACCUAUGAAGAUUACtt |
| [LOC124685](http://www.ncbi.nlm.nih.gov/entrez/query.fcgi?db=gene&cmd=search&term=LOC124685) | hCG1644301 | XM_064265 | GGACCAAGGCACCUAUGAAtt |
| [LOC124685](http://www.ncbi.nlm.nih.gov/entrez/query.fcgi?db=gene&cmd=search&term=LOC124685) | hCG1644301 | XM_064265 | AUCCCACUAACACUGAGGUtt |
| [LOC147804](http://www.ncbi.nlm.nih.gov/entrez/query.fcgi?db=gene&cmd=search&term=LOC147804) | tropomyosin 3 pseudogene | NM_001010856 | GGAAGAGAGGAAGAUUCUUtt |
| [LOC147804](http://www.ncbi.nlm.nih.gov/entrez/query.fcgi?db=gene&cmd=search&term=LOC147804) | tropomyosin 3 pseudogene | NM_001010856 | GAACCUUUGCAAACAACAUtt |
| [LOC147804](http://www.ncbi.nlm.nih.gov/entrez/query.fcgi?db=gene&cmd=search&term=LOC147804) | tropomyosin 3 pseudogene | NM_001010856 | CAAAUGUGAGGAAGAGAGGtt |
| [LOC285321](http://www.ncbi.nlm.nih.gov/entrez/query.fcgi?db=gene&cmd=search&term=LOC285321) | WITHDRAWN | XM_208313 | UUUAUUCAGCACCACACUAtt |
| [LOC285321](http://www.ncbi.nlm.nih.gov/entrez/query.fcgi?db=gene&cmd=search&term=LOC285321) | WITHDRAWN | XM_208313 | GGAAAUACACUAGCAAAUUtt |
| [LOC285321](http://www.ncbi.nlm.nih.gov/entrez/query.fcgi?db=gene&cmd=search&term=LOC285321) | WITHDRAWN | XM_208313 | GCAUAGUUUCUUAAGACAGtt |
| [LOC389031](http://www.ncbi.nlm.nih.gov/entrez/query.fcgi?db=gene&cmd=search&term=LOC389031) | WITHDRAWN | XM_371555 | GCAGCACAAGAUCUAUACAtt |
| [LOC389031](http://www.ncbi.nlm.nih.gov/entrez/query.fcgi?db=gene&cmd=search&term=LOC389031) | WITHDRAWN | XM_371555 | CCAGCUGAUGAAAAUCCUGtt |
| [LOC389031](http://www.ncbi.nlm.nih.gov/entrez/query.fcgi?db=gene&cmd=search&term=LOC389031) | WITHDRAWN | XM_371555 | CCCUGGUUUACUCCUCCAAtt |
| [LOC389765](http://www.ncbi.nlm.nih.gov/entrez/query.fcgi?db=gene&cmd=search&term=LOC389765) | kinesin family member 27 pseudogene | XM_372122 | GGACCAUCCCUACUUCAUUtt |
| [LOC389765](http://www.ncbi.nlm.nih.gov/entrez/query.fcgi?db=gene&cmd=search&term=LOC389765) | kinesin family member 27 pseudogene | XM_372122 | CGAACAAGAUCCUCAAGUCtt |
| [LOC389765](http://www.ncbi.nlm.nih.gov/entrez/query.fcgi?db=gene&cmd=search&term=LOC389765) | kinesin family member 27 pseudogene | XM_372122 | GGAGGAUUAGGAAUAUUGCtt |
| [LOC391722](http://www.ncbi.nlm.nih.gov/entrez/query.fcgi?db=gene&cmd=search&term=LOC391722) | similar to myosin regulatory light chain MRCL2 | XM_373042 | CCGGCUUUGCAUUAUUUUGtt |
| [LOC391722](http://www.ncbi.nlm.nih.gov/entrez/query.fcgi?db=gene&cmd=search&term=LOC391722) | similar to myosin regulatory light chain MRCL2 | XM_373042 | CGUCCAAGGUGUUUGCCACtt |
| [LOC391722](http://www.ncbi.nlm.nih.gov/entrez/query.fcgi?db=gene&cmd=search&term=LOC391722) | similar to myosin regulatory light chain MRCL2 | XM_373042 | GCAAGGAAGAUUUGCAUGGtt |
| [LOC391844](http://www.ncbi.nlm.nih.gov/entrez/query.fcgi?db=gene&cmd=search&term=LOC391844) | similar to tropomyosin 2 (beta), pseudogene | XM_373098 | GGAGGAAACGUGGAGAUUCtt |
| [LOC391844](http://www.ncbi.nlm.nih.gov/entrez/query.fcgi?db=gene&cmd=search&term=LOC391844) | similar to tropomyosin 2 (beta), pseudogene | XM_373098 | GGAGAUGAAACUGCUGGAGtt |
| [LOC391844](http://www.ncbi.nlm.nih.gov/entrez/query.fcgi?db=gene&cmd=search&term=LOC391844) | similar to tropomyosin 2 (beta), pseudogene | XM_373098 | CCAGAAAGACCGGGACUCAtt |
| [LOC401855](http://www.ncbi.nlm.nih.gov/entrez/query.fcgi?db=gene&cmd=search&term=LOC401855) | WITHDRAWN | XM_497509 | GCGAGAUGUUGCCAUGAUGtt |
| [LOC401855](http://www.ncbi.nlm.nih.gov/entrez/query.fcgi?db=gene&cmd=search&term=LOC401855) | WITHDRAWN | XM_497509 | GCGUUUAUGAUGGCUCUAAtt |
| [LOC401855](http://www.ncbi.nlm.nih.gov/entrez/query.fcgi?db=gene&cmd=search&term=LOC401855) | WITHDRAWN | XM_497509 | GGGCAGCAACUUUGGAUCAtt |
| [LOC440870](http://www.ncbi.nlm.nih.gov/entrez/query.fcgi?db=gene&cmd=search&term=LOC440870) | WITHDRAWN | XM_496554 | GCUUCCAGUUAUCAUUGACtt |
| [LOC440870](http://www.ncbi.nlm.nih.gov/entrez/query.fcgi?db=gene&cmd=search&term=LOC440870) | WITHDRAWN | XM_496554 | CCUCCCAGCUAAGAGAUAAtt |
| [LOC440870](http://www.ncbi.nlm.nih.gov/entrez/query.fcgi?db=gene&cmd=search&term=LOC440870) | WITHDRAWN | XM_496554 | GGAAUGGCUUGGUAAAGUGtt |
| [LOC440939](http://www.ncbi.nlm.nih.gov/entrez/query.fcgi?db=gene&cmd=search&term=LOC440939) | WITHDRAWN | XM_496620 | GGCCACGUGAAAAGAUAUUtt |
| [LOC440939](http://www.ncbi.nlm.nih.gov/entrez/query.fcgi?db=gene&cmd=search&term=LOC440939) | WITHDRAWN | XM_496620 | GCCCGUAUAAACUCGAUUCtt |
| [LOC440939](http://www.ncbi.nlm.nih.gov/entrez/query.fcgi?db=gene&cmd=search&term=LOC440939) | WITHDRAWN | XM_496620 | GCUUCAAAAAGGGAGGCAGtt |
| [LOC441930](http://www.ncbi.nlm.nih.gov/entrez/query.fcgi?db=gene&cmd=search&term=LOC441930) | WITHDRAWN | XM_497745 | GGACCACCAGGUUUUCCUAtt |
| [LOC441930](http://www.ncbi.nlm.nih.gov/entrez/query.fcgi?db=gene&cmd=search&term=LOC441930) | WITHDRAWN | XM_497745 | GGGAUAAGGAUGGUGUCCUtt |
| [LOC441930](http://www.ncbi.nlm.nih.gov/entrez/query.fcgi?db=gene&cmd=search&term=LOC441930) | WITHDRAWN | XM_497745 | CCUGGCAUAUUGGUCUCACtt |
| [LOC442057](http://www.ncbi.nlm.nih.gov/entrez/query.fcgi?db=gene&cmd=search&term=LOC442057) | WITHDRAWN | XM_497889 | CGUAGAGCCUCAAAUGCCCtt |
| [LOC442057](http://www.ncbi.nlm.nih.gov/entrez/query.fcgi?db=gene&cmd=search&term=LOC442057) | WITHDRAWN | XM_497889 | GGAAAUCUAGCUCAUCCCAtt |
| [LOC442057](http://www.ncbi.nlm.nih.gov/entrez/query.fcgi?db=gene&cmd=search&term=LOC442057) | WITHDRAWN | XM_497889 | GGUCUAGACCCGGUGAAACtt |
| [LOC442331](http://www.ncbi.nlm.nih.gov/entrez/query.fcgi?db=gene&cmd=search&term=LOC442331) | WITHDRAWN | XM_498222 | GCAUCCUCUUUUUAGAAAGtt |
| [LOC442331](http://www.ncbi.nlm.nih.gov/entrez/query.fcgi?db=gene&cmd=search&term=LOC442331) | WITHDRAWN | XM_498222 | GCAGGUAAGUCUGGUUUGGtt |
| [LOC442331](http://www.ncbi.nlm.nih.gov/entrez/query.fcgi?db=gene&cmd=search&term=LOC442331) | WITHDRAWN | XM_498222 | CCUGCUCUUGCCAGUUACUtt |
| [MRLC2](http://www.ncbi.nlm.nih.gov/entrez/query.fcgi?db=gene&cmd=search&term=MRLC2) | myosin, light chain 12B, regulatory | NM_033546 | UGACUGAAAGAACUUUAGCtt |
| [MRLC2](http://www.ncbi.nlm.nih.gov/entrez/query.fcgi?db=gene&cmd=search&term=MRLC2) | myosin, light chain 12B, regulatory | NM_033546 | CCUGUUGCAUGCAACUUAGtt |
| [MRLC2](http://www.ncbi.nlm.nih.gov/entrez/query.fcgi?db=gene&cmd=search&term=MRLC2) | myosin, light chain 12B, regulatory | NM_033546 | CCUUUCUGCCACUUAGCACtt |
| [MYH1](http://www.ncbi.nlm.nih.gov/entrez/query.fcgi?db=gene&cmd=search&term=MYH1) | myosin, heavy polypeptide 1, skeletal muscle | NM_005963 | GCUUCUCAAAAGGAAUCCCtt |
| [MYH1](http://www.ncbi.nlm.nih.gov/entrez/query.fcgi?db=gene&cmd=search&term=MYH1) | myosin, heavy polypeptide 1, skeletal muscle | NM_005963 | GGAGUCCUUUGUGAAAGCAtt |
| [MYH1](http://www.ncbi.nlm.nih.gov/entrez/query.fcgi?db=gene&cmd=search&term=MYH1) | myosin, heavy polypeptide 1, skeletal muscle | NM_005963 | GGUGAAGUUGAAAGUGAACtt |
| [MYH10](http://www.ncbi.nlm.nih.gov/entrez/query.fcgi?db=gene&cmd=search&term=MYH10) | myosin, heavy chain 10, non-muscle | NM_005964 | CAUUGAAACAUACCUUCUGtt |
| [MYH10](http://www.ncbi.nlm.nih.gov/entrez/query.fcgi?db=gene&cmd=search&term=MYH10) | myosin, heavy chain 10, non-muscle | NM_005964 | AAAGAUGAACGUACUUUUCtt |
| [MYH10](http://www.ncbi.nlm.nih.gov/entrez/query.fcgi?db=gene&cmd=search&term=MYH10) | myosin, heavy chain 10, non-muscle | NM_005964 | GCUAAAAAGCUAGUGUGGAtt |
| [MYH11](http://www.ncbi.nlm.nih.gov/entrez/query.fcgi?db=gene&cmd=search&term=MYH11) | myosin, heavy polypeptide 11, smooth m | NM_022844 | GAAGCAGAGGAAAUUUGAUtt |
| [MYH11](http://www.ncbi.nlm.nih.gov/entrez/query.fcgi?db=gene&cmd=search&term=MYH11) | myosin, heavy polypeptide 11, smooth m | NM_002474 | GGAAAUUUGAUCAGUUGUUtt |
| [MYH11](http://www.ncbi.nlm.nih.gov/entrez/query.fcgi?db=gene&cmd=search&term=MYH11) | myosin, heavy polypeptide 11, smooth m | NM_002474 | GCAUUAAGGAGGAGAAGGGtt |
| [MYH13](http://www.ncbi.nlm.nih.gov/entrez/query.fcgi?db=gene&cmd=search&term=MYH13) | myosin, heavy polypeptide 13, skeletal m | NM_003802 | CCUUGACGAAAGUUGAAAAtt |
| [MYH13](http://www.ncbi.nlm.nih.gov/entrez/query.fcgi?db=gene&cmd=search&term=MYH13) | myosin, heavy polypeptide 13, skeletal m | NM_003802 | GGUAAAGAAUCUUUCCGAAtt |
| [MYH13](http://www.ncbi.nlm.nih.gov/entrez/query.fcgi?db=gene&cmd=search&term=MYH13) | myosin, heavy polypeptide 13, skeletal m | NM_003802 | GGAAAUGUAUGUGAAAGGCtt |
| [MYH14](http://www.ncbi.nlm.nih.gov/entrez/query.fcgi?db=gene&cmd=search&term=MYH14) | myosin, heavy polypeptide 14 | NM_024729 | GCGAUACGAGAUCCUGACAtt |
| [MYH14](http://www.ncbi.nlm.nih.gov/entrez/query.fcgi?db=gene&cmd=search&term=MYH14) | myosin, heavy polypeptide 14 | NM_024729 | GGGAAACCUUCGAGUCAAGtt |
| [MYH14](http://www.ncbi.nlm.nih.gov/entrez/query.fcgi?db=gene&cmd=search&term=MYH14) | myosin, heavy polypeptide 14 | NM_024729 | GGUGACAGAACUGGAGGAUtt |
| [MYH15](http://www.ncbi.nlm.nih.gov/entrez/query.fcgi?db=gene&cmd=search&term=KIAA1000) | myosin, heavy chain 15 | XM_036988 | GCUGUCAAGGCAGUUCUUCtt |
| MYH15 | myosin, heavy chain 15 | XM_036988 | GCUCAUUUUGAACUUGUCCtt |
| MYH15 | myosin, heavy chain 15 | XM_036988 | CGUUUAGGGCCUUUUUUUGtt |
| [MYH2](http://www.ncbi.nlm.nih.gov/entrez/query.fcgi?db=gene&cmd=search&term=MYH2) | myosin, heavy polypeptide 2, skeletal muscle | NM_017534 | GGAAAAGUGACGGUGAAGAtt |
| [MYH2](http://www.ncbi.nlm.nih.gov/entrez/query.fcgi?db=gene&cmd=search&term=MYH2) | myosin, heavy polypeptide 2, skeletal muscle | NM_017534 | GGAGGAAAAGUGACGGUGAtt |
| [MYH2](http://www.ncbi.nlm.nih.gov/entrez/query.fcgi?db=gene&cmd=search&term=MYH2) | myosin, heavy polypeptide 2, skeletal muscle | NM_017534 | CGAACCCAUAUGAUUACCCtt |
| [MYH3](http://www.ncbi.nlm.nih.gov/entrez/query.fcgi?db=gene&cmd=search&term=MYH3) | myosin, heavy polypeptide 3, skeletal m | NM_002470 | GCUGAAAGCGAAAAUUUGUtt |
| [MYH3](http://www.ncbi.nlm.nih.gov/entrez/query.fcgi?db=gene&cmd=search&term=MYH3) | myosin, heavy polypeptide 3, skeletal m | NM_002470 | GGAACAAAAGGAAAUUGGAtt |
| [MYH3](http://www.ncbi.nlm.nih.gov/entrez/query.fcgi?db=gene&cmd=search&term=MYH3) | myosin, heavy polypeptide 3, skeletal m | NM_002470 | GCAUAGAUGAUCGAGAGGAtt |
| [MYH4](http://www.ncbi.nlm.nih.gov/entrez/query.fcgi?db=gene&cmd=search&term=MYH4) | myosin, heavy polypeptide 4, skeletal muscle | NM_017533 | GGAGUCCUACGUGAAAGCAtt |
| [MYH4](http://www.ncbi.nlm.nih.gov/entrez/query.fcgi?db=gene&cmd=search&term=MYH4) | myosin, heavy polypeptide 4, skeletal muscle | NM_017533 | GGCAAACUUUGAGAAAAUGtt |
| [MYH4](http://www.ncbi.nlm.nih.gov/entrez/query.fcgi?db=gene&cmd=search&term=MYH4) | myosin, heavy polypeptide 4, skeletal muscle | NM_017533 | GGAGCGAAUUGAAGCUCAGtt |
| [MYH6](http://www.ncbi.nlm.nih.gov/entrez/query.fcgi?db=gene&cmd=search&term=MYH6) | myosin, heavy polypeptide 6, cardiac muscle | NM_002471 | GGAAGAGUUUGUCAAAGCCtt |
| [MYH6](http://www.ncbi.nlm.nih.gov/entrez/query.fcgi?db=gene&cmd=search&term=MYH6) | myosin, heavy polypeptide 6, cardiac muscle | NM_002471 | GGCAAGGUCAUUGCUGAAAtt |
| [MYH6](http://www.ncbi.nlm.nih.gov/entrez/query.fcgi?db=gene&cmd=search&term=MYH6) | myosin, heavy polypeptide 6, cardiac muscle | NM_002471 | GCCAAGAUUUUGUCCCGGGtt |
| [MYH7](http://www.ncbi.nlm.nih.gov/entrez/query.fcgi?db=gene&cmd=search&term=MYH7) | myosin, heavy chain 7, cardiac muscle | NM_000257 | GGCCUUUUGACCUCAAGAAtt |
| [MYH7](http://www.ncbi.nlm.nih.gov/entrez/query.fcgi?db=gene&cmd=search&term=MYH7) | myosin, heavy chain 7, cardiac muscle | NM_000257 | GGAAGCAGAAUAAAGCAAUtt |
| [MYH7](http://www.ncbi.nlm.nih.gov/entrez/query.fcgi?db=gene&cmd=search&term=MYH7) | myosin, heavy chain 7, cardiac muscle | NM_000257 | GGGCUUGAAUGAGGAGUAGtt |
| [MYH7B](http://www.ncbi.nlm.nih.gov/entrez/query.fcgi?db=gene&cmd=search&term=MYH7B) | myosin, heavy chain 7B, cardiac muscle | NM_020884 | GGAGCAAGUACGAAGCAGAtt |
| [MYH7B](http://www.ncbi.nlm.nih.gov/entrez/query.fcgi?db=gene&cmd=search&term=MYH7B) | myosin, heavy chain 7B, cardiac muscle | NM_020884 | AGAACUGGUCAUGGAUGAAtt |
| [MYH7B](http://www.ncbi.nlm.nih.gov/entrez/query.fcgi?db=gene&cmd=search&term=MYH7B) | myosin, heavy chain 7B, cardiac muscle | NM_020884 | ACACCAAGCGGGUCAUUCAtt |
| [MYH8](http://www.ncbi.nlm.nih.gov/entrez/query.fcgi?db=gene&cmd=search&term=MYH8) | myosin, heavy polypeptide 8, skeletal m | NM_002472 | GGUUGAAAAUGAACAGAAAtt |
| [MYH8](http://www.ncbi.nlm.nih.gov/entrez/query.fcgi?db=gene&cmd=search&term=MYH8) | myosin, heavy polypeptide 8, skeletal m | NM_002472 | GGAAUCCUAUGUGAAGAGCtt |
| [MYH8](http://www.ncbi.nlm.nih.gov/entrez/query.fcgi?db=gene&cmd=search&term=MYH8) | myosin, heavy polypeptide 8, skeletal m | NM_002472 | GCAGAAUCUUAUAUGGUGAtt |
| [MYH9](http://www.ncbi.nlm.nih.gov/entrez/query.fcgi?db=gene&cmd=search&term=MYH9) | myosin, heavy polypeptide 9, non-muscle | NM_002473 | GGUGAAGGUGAACAAGGAUtt |
| [MYH9](http://www.ncbi.nlm.nih.gov/entrez/query.fcgi?db=gene&cmd=search&term=MYH9) | myosin, heavy polypeptide 9, non-muscle | NM_002473 | GCAUGAGGCAAUGAUCACUtt |
| [MYH9](http://www.ncbi.nlm.nih.gov/entrez/query.fcgi?db=gene&cmd=search&term=MYH9) | myosin, heavy polypeptide 9, non-muscle | NM_002473 | CCGCCUACAGGAGUAUGAUtt |
| [MYL1](http://www.ncbi.nlm.nih.gov/entrez/query.fcgi?db=gene&cmd=search&term=MYL1) | myosin, light polypeptide 1, alkali; skeletal | NM_079420 | GCAUUGUUUAGGAAGACUGtt |
| [MYL1](http://www.ncbi.nlm.nih.gov/entrez/query.fcgi?db=gene&cmd=search&term=MYL1) | myosin, light polypeptide 1, alkali; skeletal | NM_079420 | GGUCAGGAAAGUUCUGGGAtt |
| [MYL1](http://www.ncbi.nlm.nih.gov/entrez/query.fcgi?db=gene&cmd=search&term=MYL1) | myosin, light polypeptide 1, alkali; skeletal | NM_079420 | GGAAAGUUCUGGGAAACCCtt |
| [MYL12A](http://www.ncbi.nlm.nih.gov/entrez/query.fcgi?db=gene&cmd=search&term=MRCL3) | myosin, light chain 12A, regulatory | NM_006471 | GCCAUUUUGGGCAUAUGUAtt |
| MYL12A | myosin, light chain 12A, regulatory | NM_006471 | GAAUCCAACUGAUGAGUAUtt |
| MYL12A | myosin, light chain 12A, regulatory | NM_006471 | GCUUUUGCAUUUCCUGUUGtt |
| [MYL2](http://www.ncbi.nlm.nih.gov/entrez/query.fcgi?db=gene&cmd=search&term=MYL2) | myosin, light polypeptide 2, regulatory, cardiac | NM_000432 | GGCCAUGUUCCAAUAAACAtt |
| [MYL2](http://www.ncbi.nlm.nih.gov/entrez/query.fcgi?db=gene&cmd=search&term=MYL2) | myosin, light polypeptide 2, regulatory, cardiac | NM_000432 | GGAGGUCGUGUAUUUGGUCtt |
| [MYL2](http://www.ncbi.nlm.nih.gov/entrez/query.fcgi?db=gene&cmd=search&term=MYL2) | myosin, light polypeptide 2, regulatory, cardiac | NM_000432 | GGGAUGGCUUCAUUGACAAtt |
| [MYL3](http://www.ncbi.nlm.nih.gov/entrez/query.fcgi?db=gene&cmd=search&term=MYL3) | myosin, light polypeptide 3, alkali | NM_000258 | GGAAGAGCUCAAUACCAAGtt |
| [MYL3](http://www.ncbi.nlm.nih.gov/entrez/query.fcgi?db=gene&cmd=search&term=MYL3) | myosin, light polypeptide 3, alkali | NM_000258 | GGAGGUCGAGUUUGAUGCUtt |
| [MYL3](http://www.ncbi.nlm.nih.gov/entrez/query.fcgi?db=gene&cmd=search&term=MYL3) | myosin, light polypeptide 3, alkali | NM_000258 | GGAAGCCUUCAUGCUGUUCtt |
| [MYL4](http://www.ncbi.nlm.nih.gov/entrez/query.fcgi?db=gene&cmd=search&term=MYL4) | myosin, light polypeptide 4, embryonic | NM_002476 | GGCCUUUUCAUUGUUUGACtt |
| [MYL4](http://www.ncbi.nlm.nih.gov/entrez/query.fcgi?db=gene&cmd=search&term=MYL4) | myosin, light polypeptide 4, embryonic | NM_001002841 | GGCCCUCCCUGUUAAUAAAtt |
| [MYL4](http://www.ncbi.nlm.nih.gov/entrez/query.fcgi?db=gene&cmd=search&term=MYL4) | myosin, light polypeptide 4, embryonic | NM_001002841 | GCACAUCAUGUCAGGGUGAtt |
| [MYL5](http://www.ncbi.nlm.nih.gov/entrez/query.fcgi?db=gene&cmd=search&term=MYL5) | myosin, light polypeptide 5, regulatory | NM_002477 | GGGAAAAUCAACAAGGAGUtt |
| [MYL5](http://www.ncbi.nlm.nih.gov/entrez/query.fcgi?db=gene&cmd=search&term=MYL5) | myosin, light polypeptide 5, regulatory | NM_002477 | GGAGUUCAAGGAGGCAUUCtt |
| [MYL5](http://www.ncbi.nlm.nih.gov/entrez/query.fcgi?db=gene&cmd=search&term=MYL5) | myosin, light polypeptide 5, regulatory | NM_002477 | GGAGGCAUUCACACUCAUGtt |
| [MYL6](http://www.ncbi.nlm.nih.gov/entrez/query.fcgi?db=gene&cmd=search&term=custom) | myosin, light chain 6 | AB046613 | UGAGAUGAAUGUGAAGGUGtt |
| MYL6 | myosin, light chain 6 | AB046613 | GCAUUCACCAAAUAAACUUtt |
| MYL6 | myosin, light chain 6 | AB046613 | CUUUGAGCACUUUCUGCCCtt |
| [MYL6B](http://www.ncbi.nlm.nih.gov/entrez/query.fcgi?db=gene&cmd=search&term=MLC1SA) | myosin, light chain 6B | NM_002475 | GGCCACCUAUUGUUUCAAAtt |
| MYL6B | myosin, light chain 6B | NM_002475 | GGCACAUAUGAGGACUACUtt |
| MYL6B | myosin, light chain 6B | NM_002475 | GCUCAGACAUGUUCUCACCtt |
| [MYL7](http://www.ncbi.nlm.nih.gov/entrez/query.fcgi?db=gene&cmd=search&term=MYL7) | myosin, light polypeptide 7, regulatory | NM_021223 | GGCAGACAAGUUCUCUCCAtt |
| [MYL7](http://www.ncbi.nlm.nih.gov/entrez/query.fcgi?db=gene&cmd=search&term=MYL7) | myosin, light polypeptide 7, regulatory | NM_021223 | CCUCAAUAAACUCUGUUGCtt |
| [MYL7](http://www.ncbi.nlm.nih.gov/entrez/query.fcgi?db=gene&cmd=search&term=MYL7) | myosin, light polypeptide 7, regulatory | NM_021223 | GGAUGAGUUCAAGCAGCUUtt |
| [MYL8P](http://www.ncbi.nlm.nih.gov/entrez/query.fcgi?db=gene&cmd=search&term=LOC442204) | myosin, light chain 8, pseudogene | XM_498088 | GGAGAGUUUGUGCAAAUUGtt |
| [MYL8P](http://www.ncbi.nlm.nih.gov/entrez/query.fcgi?db=gene&cmd=search&term=LOC442204) | myosin, light chain 8, pseudogene | XM_498088 | GCCAGUGGAGCUCUUUUCAtt |
| [MYL8P](http://www.ncbi.nlm.nih.gov/entrez/query.fcgi?db=gene&cmd=search&term=LOC442204) | myosin, light chain 8, pseudogene | XM_498088 | CCGGAAGUCACAGAGUUUCtt |
| [MYL9](http://www.ncbi.nlm.nih.gov/entrez/query.fcgi?db=gene&cmd=search&term=MYL9) | myosin, light chain 9, regulatory | NM_006097 | GGCUUUCAACAUGAUUGACtt |
| [MYL9](http://www.ncbi.nlm.nih.gov/entrez/query.fcgi?db=gene&cmd=search&term=MYL9) | myosin, light chain 9, regulatory | NM_181526 | GGAGGCUUUCAACAUGAUUtt |
| [MYL9](http://www.ncbi.nlm.nih.gov/entrez/query.fcgi?db=gene&cmd=search&term=MYL9) | myosin, light chain 9, regulatory | NM_181526 | CCCAUUGAUAAGAAAGGCAtt |
| [MYLC2PL](http://www.ncbi.nlm.nih.gov/entrez/query.fcgi?db=gene&cmd=search&term=MYLC2PL) | myosin, light chain 10, regulatory | NM_138403 | GGCCGAUGUCAUCAAAGAAtt |
| [MYLC2PL](http://www.ncbi.nlm.nih.gov/entrez/query.fcgi?db=gene&cmd=search&term=MYLC2PL) | myosin, light chain 10, regulatory | NM_138403 | GGGAAAGGUUUCGUCAAGGtt |
| [MYLC2PL](http://www.ncbi.nlm.nih.gov/entrez/query.fcgi?db=gene&cmd=search&term=MYLC2PL) | myosin, light chain 10, regulatory | NM_138403 | GGUUUCGUCAAGGCCGAUGtt |
| [MYLPF](http://www.ncbi.nlm.nih.gov/entrez/query.fcgi?db=gene&cmd=search&term=HUMMLC2B) | myosin light chain, phosphorylatable | NM_013292 | GGGCACCAUCAAGAAGAAGtt |
| MYLPF | myosin light chain, phosphorylatable | NM_013292 | GGAGGAGAUCAAGAACAUGtt |
| MYLPF | myosin light chain, phosphorylatable | NM_013292 | GGAGUUGGAUGCCAUGAUGtt |
| [MYO10](http://www.ncbi.nlm.nih.gov/entrez/query.fcgi?db=gene&cmd=search&term=MYO10) | myosin X | NM_012334 | GCCGAUUUGACUUUAUCUAtt |
| [MYO10](http://www.ncbi.nlm.nih.gov/entrez/query.fcgi?db=gene&cmd=search&term=MYO10) | myosin X | NM_012334 | GGAGAAGACAUCCUGUGUUtt |
| [MYO10](http://www.ncbi.nlm.nih.gov/entrez/query.fcgi?db=gene&cmd=search&term=MYO10) | myosin X | NM_012334 | GGUAUUCACUUACAAGCAGtt |
| [MYO15A](http://www.ncbi.nlm.nih.gov/entrez/query.fcgi?db=gene&cmd=search&term=MYO15A) | myosin XVA | NM_016239 | CGUCAAAGCUCAUGACGCAtt |
| [MYO15A](http://www.ncbi.nlm.nih.gov/entrez/query.fcgi?db=gene&cmd=search&term=MYO15A) | myosin XVA | NM_016239 | GGACCUGGGCGAGUAUUAUtt |
| [MYO15A](http://www.ncbi.nlm.nih.gov/entrez/query.fcgi?db=gene&cmd=search&term=MYO15A) | myosin XVA | NM_016239 | GGUGCCCUAUUUUUACCCGtt |
| [MYO15B](http://www.ncbi.nlm.nih.gov/entrez/query.fcgi?db=gene&cmd=search&term=MYO15B) | myosin XVB pseudogene | BC027875 | CCGGGUGAAAUGAAGGCUUtt |
| [MYO15B](http://www.ncbi.nlm.nih.gov/entrez/query.fcgi?db=gene&cmd=search&term=MYO15B) | myosin XVB pseudogene | BC027875 | CCACUGUCUUCCUGAUAGAtt |
| [MYO15B](http://www.ncbi.nlm.nih.gov/entrez/query.fcgi?db=gene&cmd=search&term=MYO15B) | myosin XVB pseudogene | BC027875 | CGAAUAUCCAGACUUUCACtt |
| [MYO16](http://www.ncbi.nlm.nih.gov/entrez/query.fcgi?db=gene&cmd=search&term=MYR8) | myosin XVI | NM_015011 | UCACAACUCAGGAAAUCACtt |
| [MYO16](http://www.ncbi.nlm.nih.gov/entrez/query.fcgi?db=gene&cmd=search&term=MYR8) | myosin XVI | NM_015011 | UUUCCUGUCAAGGUAUAAGtt |
| [MYO16](http://www.ncbi.nlm.nih.gov/entrez/query.fcgi?db=gene&cmd=search&term=MYR8) | myosin XVI | NM_015011 | GGCCCAAUAACUCAAAGCUtt |
| [MYO18A](http://www.ncbi.nlm.nih.gov/entrez/query.fcgi?db=gene&cmd=search&term=MYO18A) | myosin XVIIIA | NM_203318 | GGAUCAGUCAAUCAUCCUCtt |
| [MYO18A](http://www.ncbi.nlm.nih.gov/entrez/query.fcgi?db=gene&cmd=search&term=MYO18A) | myosin XVIIIA | NM_078471 | GGAGUUGGAAAGAUACAAGtt |
| [MYO18A](http://www.ncbi.nlm.nih.gov/entrez/query.fcgi?db=gene&cmd=search&term=MYO18A) | myosin XVIIIA | NM_203318 | GCCAUCCGAUGCGAAAACAtt |
| [MYO18B](http://www.ncbi.nlm.nih.gov/entrez/query.fcgi?db=gene&cmd=search&term=MYO18B) | myosin XVIIIB | NM_032608 | GCAUAAUGAAGAAAUACCUtt |
| [MYO18B](http://www.ncbi.nlm.nih.gov/entrez/query.fcgi?db=gene&cmd=search&term=MYO18B) | myosin XVIIIB | NM_032608 | GGAACUGUGGCACUGAAAAtt |
| [MYO18B](http://www.ncbi.nlm.nih.gov/entrez/query.fcgi?db=gene&cmd=search&term=MYO18B) | myosin XVIIIB | NM_032608 | GGUGCAGAUUAAGAGAUUUtt |
| [MYO1A](http://www.ncbi.nlm.nih.gov/entrez/query.fcgi?db=gene&cmd=search&term=MYO1A) | myosin IA | NM_005379 | GGUGGACUACUUUGAUAAUtt |
| [MYO1A](http://www.ncbi.nlm.nih.gov/entrez/query.fcgi?db=gene&cmd=search&term=MYO1A) | myosin IA | NM_005379 | GGAGAGCAGGUGAACUCUGtt |
| [MYO1A](http://www.ncbi.nlm.nih.gov/entrez/query.fcgi?db=gene&cmd=search&term=MYO1A) | myosin IA | NM_005379 | GCUUGAGCGGGAUACAACUtt |
| [MYO1B](http://www.ncbi.nlm.nih.gov/entrez/query.fcgi?db=gene&cmd=search&term=MYO1B) | myosin IB | NM_012223 | CCCAUACCGGUCUUUACCCtt |
| [MYO1B](http://www.ncbi.nlm.nih.gov/entrez/query.fcgi?db=gene&cmd=search&term=MYO1B) | myosin IB | NM_012223 | CCCAAACUAUAUUAGGUGUtt |
| [MYO1B](http://www.ncbi.nlm.nih.gov/entrez/query.fcgi?db=gene&cmd=search&term=MYO1B) | myosin IB | NM_012223 | CCGGCAUUGAUCAAUCAGUtt |
| [MYO1C](http://www.ncbi.nlm.nih.gov/entrez/query.fcgi?db=gene&cmd=search&term=MYO1C) | myosin IC | NM_033375 | GGGCAAGAAGGAUAAUUACtt |
| [MYO1C](http://www.ncbi.nlm.nih.gov/entrez/query.fcgi?db=gene&cmd=search&term=MYO1C) | myosin IC | NM_033375 | GCCAGAAGAGUACAAGAUGtt |
| [MYO1C](http://www.ncbi.nlm.nih.gov/entrez/query.fcgi?db=gene&cmd=search&term=MYO1C) | myosin IC | NM_033375 | GGAGACCAUGUGUAGCUCAtt |
| [MYO1D](http://www.ncbi.nlm.nih.gov/entrez/query.fcgi?db=gene&cmd=search&term=MYO1D) | myosin ID | NM_015194 | GGCAAACUGAGCAUUACAGtt |
| [MYO1D](http://www.ncbi.nlm.nih.gov/entrez/query.fcgi?db=gene&cmd=search&term=MYO1D) | myosin ID | NM_015194 | GGAUCAUCUUACUUAACUCtt |
| [MYO1D](http://www.ncbi.nlm.nih.gov/entrez/query.fcgi?db=gene&cmd=search&term=MYO1D) | myosin ID | NM_015194 | AAUGCUACGCUCUCUACAUtt |
| [MYO1E](http://www.ncbi.nlm.nih.gov/entrez/query.fcgi?db=gene&cmd=search&term=MYO1E) | myosin IE | NM_004998 | GCACGUGAAGGACAUUAUCtt |
| [MYO1E](http://www.ncbi.nlm.nih.gov/entrez/query.fcgi?db=gene&cmd=search&term=MYO1E) | myosin IE | NM_004998 | GGAUGACAUUUUUAUUCUCtt |
| [MYO1E](http://www.ncbi.nlm.nih.gov/entrez/query.fcgi?db=gene&cmd=search&term=MYO1E) | myosin IE | NM_004998 | CCCAUUGAGUACUUUAAUAtt |
| [MYO1F](http://www.ncbi.nlm.nih.gov/entrez/query.fcgi?db=gene&cmd=search&term=MYO1F) | myosin IF | NM_012335 | GGUCCAGCACGUCAAAGAUtt |
| [MYO1F](http://www.ncbi.nlm.nih.gov/entrez/query.fcgi?db=gene&cmd=search&term=MYO1F) | myosin IF | NM_012335 | GCACGUCAAAGAUAUCAUCtt |
| [MYO1F](http://www.ncbi.nlm.nih.gov/entrez/query.fcgi?db=gene&cmd=search&term=MYO1F) | myosin IF | NM_012335 | CCGUGAGAUCGACCUCUAUtt |
| [MYO1G](http://www.ncbi.nlm.nih.gov/entrez/query.fcgi?db=gene&cmd=search&term=MYO1G) | myosin IG | NM_033054 | GGUGUUUCCCGUCAACAGUtt |
| [MYO1G](http://www.ncbi.nlm.nih.gov/entrez/query.fcgi?db=gene&cmd=search&term=MYO1G) | myosin IG | NM_033054 | GGCAGGGAACUCAUAGAGAtt |
| [MYO1G](http://www.ncbi.nlm.nih.gov/entrez/query.fcgi?db=gene&cmd=search&term=MYO1G) | myosin IG | NM_033054 | GGCCCUGAGUAUGGCAAACtt |
| [MYO3A](http://www.ncbi.nlm.nih.gov/entrez/query.fcgi?db=gene&cmd=search&term=MYO3A) | myosin IIIA | NM_017433 | CCUGAAAUCACAAUACUUCtt |
| [MYO3A](http://www.ncbi.nlm.nih.gov/entrez/query.fcgi?db=gene&cmd=search&term=MYO3A) | myosin IIIA | NM_017433 | GGGCAAAGAUGUGAUGCUAtt |
| [MYO3A](http://www.ncbi.nlm.nih.gov/entrez/query.fcgi?db=gene&cmd=search&term=MYO3A) | myosin IIIA | NM_017433 | CGAUAUUGACGAAGAGAUUtt |
| [MYO3B](http://www.ncbi.nlm.nih.gov/entrez/query.fcgi?db=gene&cmd=search&term=MYO3B) | myosin IIIB | NM_138995 | GGCACCUAUGGCAAAGUCUtt |
| [MYO3B](http://www.ncbi.nlm.nih.gov/entrez/query.fcgi?db=gene&cmd=search&term=MYO3B) | myosin IIIB | NM_138995 | GGAAUGUUACCUUCAAUUGtt |
| [MYO3B](http://www.ncbi.nlm.nih.gov/entrez/query.fcgi?db=gene&cmd=search&term=MYO3B) | myosin IIIB | NM_138995 | GGAGUUAAGCUCGUUGACUtt |
| [MYO5A](http://www.ncbi.nlm.nih.gov/entrez/query.fcgi?db=gene&cmd=search&term=MYO5A) | myosin VA (heavy polypeptide 12, myoxin) | NM_000259 | GCUGCUCAAAGAUUAUAAGtt |
| [MYO5A](http://www.ncbi.nlm.nih.gov/entrez/query.fcgi?db=gene&cmd=search&term=MYO5A) | myosin VA (heavy polypeptide 12, myoxin) | NM_000259 | GGAAGGAAAGGAUUUGGAAtt |
| [MYO5A](http://www.ncbi.nlm.nih.gov/entrez/query.fcgi?db=gene&cmd=search&term=MYO5A) | myosin VA (heavy polypeptide 12, myoxin) | NM_000259 | GGAGAUAAAGUCCUCCUGCtt |
| [MYO5B](http://www.ncbi.nlm.nih.gov/entrez/query.fcgi?db=gene&cmd=search&term=MYO5B) | myosin 5B | XM_371116 | CCUUGUGAAGGAAUAUUCAtt |
| [MYO5B](http://www.ncbi.nlm.nih.gov/entrez/query.fcgi?db=gene&cmd=search&term=MYO5B) | myosin 5B | XM_371116 | GCUCAACAACCAAAUCCUGtt |
| [MYO5B](http://www.ncbi.nlm.nih.gov/entrez/query.fcgi?db=gene&cmd=search&term=MYO5B) | myosin 5B | XM_371116 | GCAGGAGAGGAAAAAGCUGtt |
| [MYO5C](http://www.ncbi.nlm.nih.gov/entrez/query.fcgi?db=gene&cmd=search&term=MYO5C) | myosin VC | NM_018728 | GCUAAGUGAAGGUUCAAUAtt |
| [MYO5C](http://www.ncbi.nlm.nih.gov/entrez/query.fcgi?db=gene&cmd=search&term=MYO5C) | myosin VC | NM_018728 | GCCAACAUGAGCACUUACCtt |
| [MYO5C](http://www.ncbi.nlm.nih.gov/entrez/query.fcgi?db=gene&cmd=search&term=MYO5C) | myosin VC | NM_018728 | GGACUACAGAGUUGGUGACtt |
| [MYO6](http://www.ncbi.nlm.nih.gov/entrez/query.fcgi?db=gene&cmd=search&term=MYO6) | myosin VI | NM_004999 | GGUUUAGGUGUUAAUGAAGtt |
| [MYO6](http://www.ncbi.nlm.nih.gov/entrez/query.fcgi?db=gene&cmd=search&term=MYO6) | myosin VI | NM_004999 | GGCAAGACAUUUUUGGCUCtt |
| [MYO6](http://www.ncbi.nlm.nih.gov/entrez/query.fcgi?db=gene&cmd=search&term=MYO6) | myosin VI | NM_004999 | CCCUACAGAUGGAUUUCAGtt |
| [MYO7A](http://www.ncbi.nlm.nih.gov/entrez/query.fcgi?db=gene&cmd=search&term=MYO7A) | myosin VIIA | NM_000260 | GGAACUACCACGUGUUCUAtt |
| [MYO7A](http://www.ncbi.nlm.nih.gov/entrez/query.fcgi?db=gene&cmd=search&term=MYO7A) | myosin VIIA | NM_000260 | GGUGGUGGAUGAUGAAGACtt |
| [MYO7A](http://www.ncbi.nlm.nih.gov/entrez/query.fcgi?db=gene&cmd=search&term=MYO7A) | myosin VIIA | NM_000260 | CGCAACGCACAUCAAGCCUtt |
| [MYO7B](http://www.ncbi.nlm.nih.gov/entrez/query.fcgi?db=gene&cmd=search&term=MYO7B) | myosin VIIB | XM_291001 | GGAGAGGUCCAUUUUCGCCtt |
| [MYO7B](http://www.ncbi.nlm.nih.gov/entrez/query.fcgi?db=gene&cmd=search&term=MYO7B) | myosin VIIB | XM_291001 | GGAGUACGAGUUUGUGUCAtt |
| [MYO7B](http://www.ncbi.nlm.nih.gov/entrez/query.fcgi?db=gene&cmd=search&term=MYO7B) | myosin VIIB | XM_291001 | CCUGCUGACCUCAUAUGUGtt |
| [MYO9A](http://www.ncbi.nlm.nih.gov/entrez/query.fcgi?db=gene&cmd=search&term=MYO9A) | myosin IXA | NM_006901 | GGUGAUUGAGUCUCUUAUAtt |
| [MYO9A](http://www.ncbi.nlm.nih.gov/entrez/query.fcgi?db=gene&cmd=search&term=MYO9A) | myosin IXA | NM_006901 | GGAUUUCCGGGAAAAAAAUtt |
| [MYO9A](http://www.ncbi.nlm.nih.gov/entrez/query.fcgi?db=gene&cmd=search&term=MYO9A) | myosin IXA | NM_006901 | GGAUUUGCCAGUGGAGUAGtt |
| [MYO9B](http://www.ncbi.nlm.nih.gov/entrez/query.fcgi?db=gene&cmd=search&term=MYO9B) | myosin IXB | NM_004145 | GGUGAAAUAUCAGAUCAAGtt |
| [MYO9B](http://www.ncbi.nlm.nih.gov/entrez/query.fcgi?db=gene&cmd=search&term=MYO9B) | myosin IXB | NM_004145 | GGUGAAGCGAGAAAUCUUGtt |
| [MYO9B](http://www.ncbi.nlm.nih.gov/entrez/query.fcgi?db=gene&cmd=search&term=MYO9B) | myosin IXB | NM_004145 | GGAGCUGUCGUCGAGAAAUtt |
| [MYRIP](http://www.ncbi.nlm.nih.gov/entrez/query.fcgi?db=gene&cmd=search&term=MYRIP) | myosin VIIA and Rab interacting protein | NM_015460 | GGUUCUGAAGAACCUGUACtt |
| [MYRIP](http://www.ncbi.nlm.nih.gov/entrez/query.fcgi?db=gene&cmd=search&term=MYRIP) | myosin VIIA and Rab interacting protein | NM_015460 | GGACAAACUGUGCAUUUAAtt |
| [MYRIP](http://www.ncbi.nlm.nih.gov/entrez/query.fcgi?db=gene&cmd=search&term=MYRIP) | myosin VIIA and Rab interacting protein | NM_015460 | GGACAUAGUGUGAUGGACAtt |
| [TEX9](http://www.ncbi.nlm.nih.gov/entrez/query.fcgi?db=gene&cmd=search&term=LOC161577) | testis expressed 9 | NM_198524 | GGCCAAACUCCAUGUUAUGtt |
| TEX9 | testis expressed 9 | NM_198524 | CGAAGAUGAUUACAGUUUAtt |
| TEX9 | testis expressed 9 | NM_198524 | GCUGACGUGGUUCAACAAGtt |
| [TNNT1](http://www.ncbi.nlm.nih.gov/entrez/query.fcgi?db=gene&cmd=search&term=TNNT1) | troponin T1, skeletal, slow | NM_003283 | GGAUGAUGCCAAGAAAAAGtt |
| [TNNT1](http://www.ncbi.nlm.nih.gov/entrez/query.fcgi?db=gene&cmd=search&term=TNNT1) | troponin T1, skeletal, slow | NM_003283 | GGAGAAGAUGAGGAAGGAAtt |
| [TNNT1](http://www.ncbi.nlm.nih.gov/entrez/query.fcgi?db=gene&cmd=search&term=TNNT1) | troponin T1, skeletal, slow | NM_003283 | GGAGGAAGAGGAGCUGGUUtt |
| [TNNT2](http://www.ncbi.nlm.nih.gov/entrez/query.fcgi?db=gene&cmd=search&term=TNNT2) | troponin T2, cardiac | NM_001001432 | GGCUUUGUCCAACAUGAUGtt |
| [TNNT2](http://www.ncbi.nlm.nih.gov/entrez/query.fcgi?db=gene&cmd=search&term=TNNT2) | troponin T2, cardiac | NM_001001431 | GGCUCACUUUGAGAACAGGtt |
| [TNNT2](http://www.ncbi.nlm.nih.gov/entrez/query.fcgi?db=gene&cmd=search&term=TNNT2) | troponin T2, cardiac | NM_001001431 | GGUCGUUCAUGCCCAACUUtt |
| [TNNT3](http://www.ncbi.nlm.nih.gov/entrez/query.fcgi?db=gene&cmd=search&term=TNNT3) | troponin T3, skeletal, fast | NM_006757 | GGAAGUUCAAGAAGACACCtt |
| [TNNT3](http://www.ncbi.nlm.nih.gov/entrez/query.fcgi?db=gene&cmd=search&term=TNNT3) | troponin T3, skeletal, fast | NM_006757 | GGAUUCGUGCAGAGAAGGAtt |
| [TNNT3](http://www.ncbi.nlm.nih.gov/entrez/query.fcgi?db=gene&cmd=search&term=TNNT3) | troponin T3, skeletal, fast | NM_006757 | GGAGAAACCGAGACCCAAAtt |
| [TPM1](http://www.ncbi.nlm.nih.gov/entrez/query.fcgi?db=gene&cmd=search&term=custom) | tropomyosin 1 (alpha) | NM_001018004 | ACAGCUUUGCAGAAGCUGGtt |
| TPM1 | tropomyosin 1 (alpha) | NM_001018004 | GUGAGAGAGGCAUGAAAGUtt |
| TPM1 | tropomyosin 1 (alpha) | NM_001018004 | AAAAAUGGAAAUUCAGGAGtt |
| [TPM2](http://www.ncbi.nlm.nih.gov/entrez/query.fcgi?db=gene&cmd=search&term=TPM2) | tropomyosin 2 (beta) | NM_003289 | GGAUGAGGUGGAAAAGUAUtt |
| [TPM2](http://www.ncbi.nlm.nih.gov/entrez/query.fcgi?db=gene&cmd=search&term=TPM2) | tropomyosin 2 (beta) | NM_213674 | GGAAUGAAGGUCAUCGAAAtt |
| [TPM2](http://www.ncbi.nlm.nih.gov/entrez/query.fcgi?db=gene&cmd=search&term=TPM2) | tropomyosin 2 (beta) | NM_003289 | GGUGGAAAAGUAUUCUGAAtt |
| [TPM3](http://www.ncbi.nlm.nih.gov/entrez/query.fcgi?db=gene&cmd=search&term=TPM3) | tropomyosin 3 | NM_153649 | GCUGGAAGAAGCUGAAAAAtt |
| [TPM3](http://www.ncbi.nlm.nih.gov/entrez/query.fcgi?db=gene&cmd=search&term=TPM3) | tropomyosin 3 | NM_152263 | GGUAUGAAGGUUAUUGAAAtt |
| [TPM3](http://www.ncbi.nlm.nih.gov/entrez/query.fcgi?db=gene&cmd=search&term=TPM3) | tropomyosin 3 | NM_152263 | GGAAGAAAUCAAGAUUCUUtt |
| [TPM4](http://www.ncbi.nlm.nih.gov/entrez/query.fcgi?db=gene&cmd=search&term=TPM4) | tropomyosin 4 | NM_003290 | GGAAUGAAGGUGAUAGAAAtt |
| [TPM4](http://www.ncbi.nlm.nih.gov/entrez/query.fcgi?db=gene&cmd=search&term=TPM4) | tropomyosin 4 | NM_003290 | GCCAAUAAAAGGACUGGUGtt |
| [TPM4](http://www.ncbi.nlm.nih.gov/entrez/query.fcgi?db=gene&cmd=search&term=TPM4) | tropomyosin 4 | NM_003290 | GGUGAUAGAAAACCGGGCCtt |
