# Supplementary figures and images for: Identification of Cytoskeleton-Associated Proteins Essential for Lysosomal Stability and Survival of Human Cancer Cells
Source: PLoS One. 2012 Oct 11;7(10):e45381. doi: 10.1371/journal.pone.0045381 (PMC3469574; doi:10.1371/journal.pone.0045381)

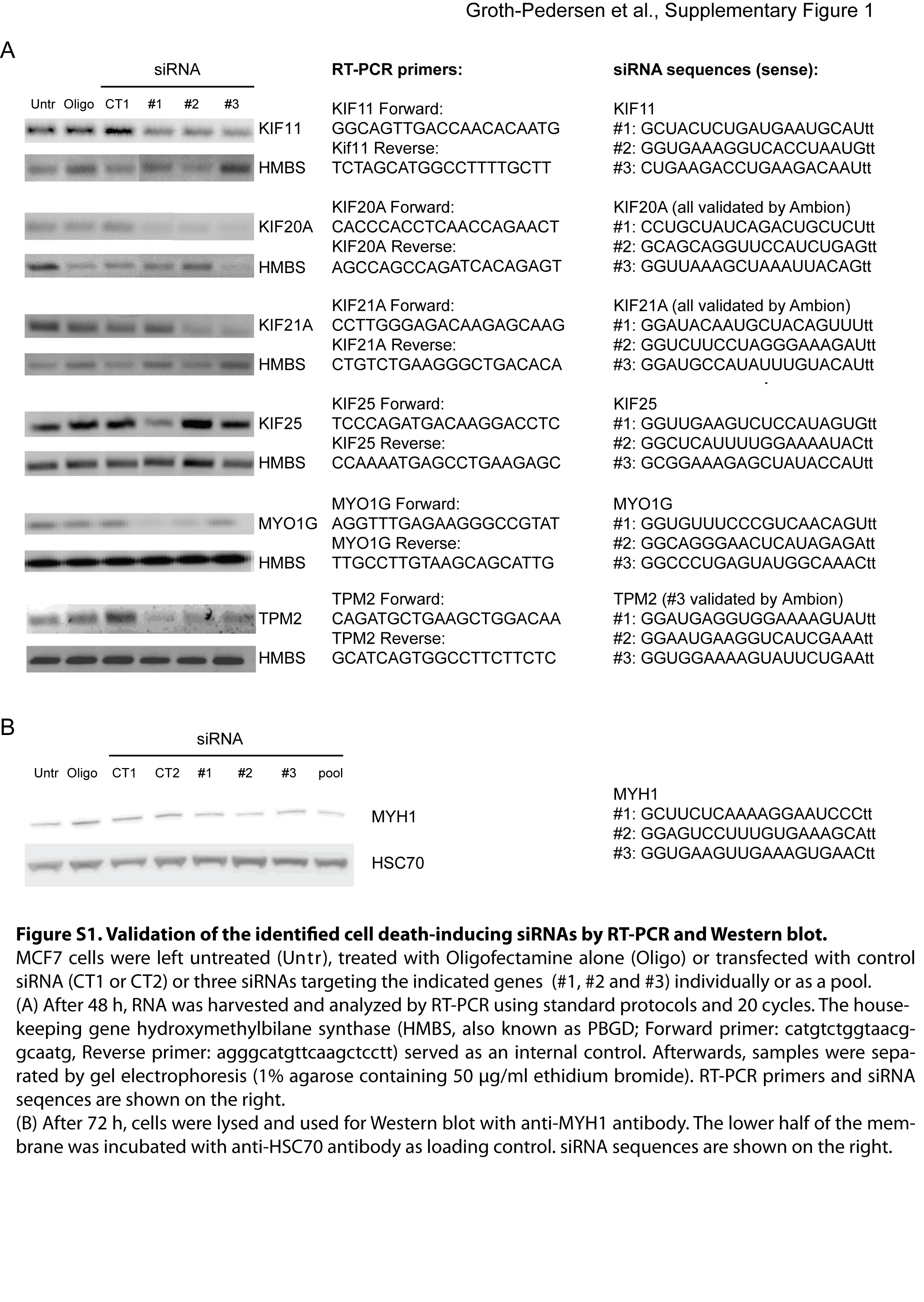

Supplement: Figure S1 — Validation of the identified cell death-inducing siRNAs by RT-PCR and Western blot. (TIF) [file pone.0045381.s002.tif]

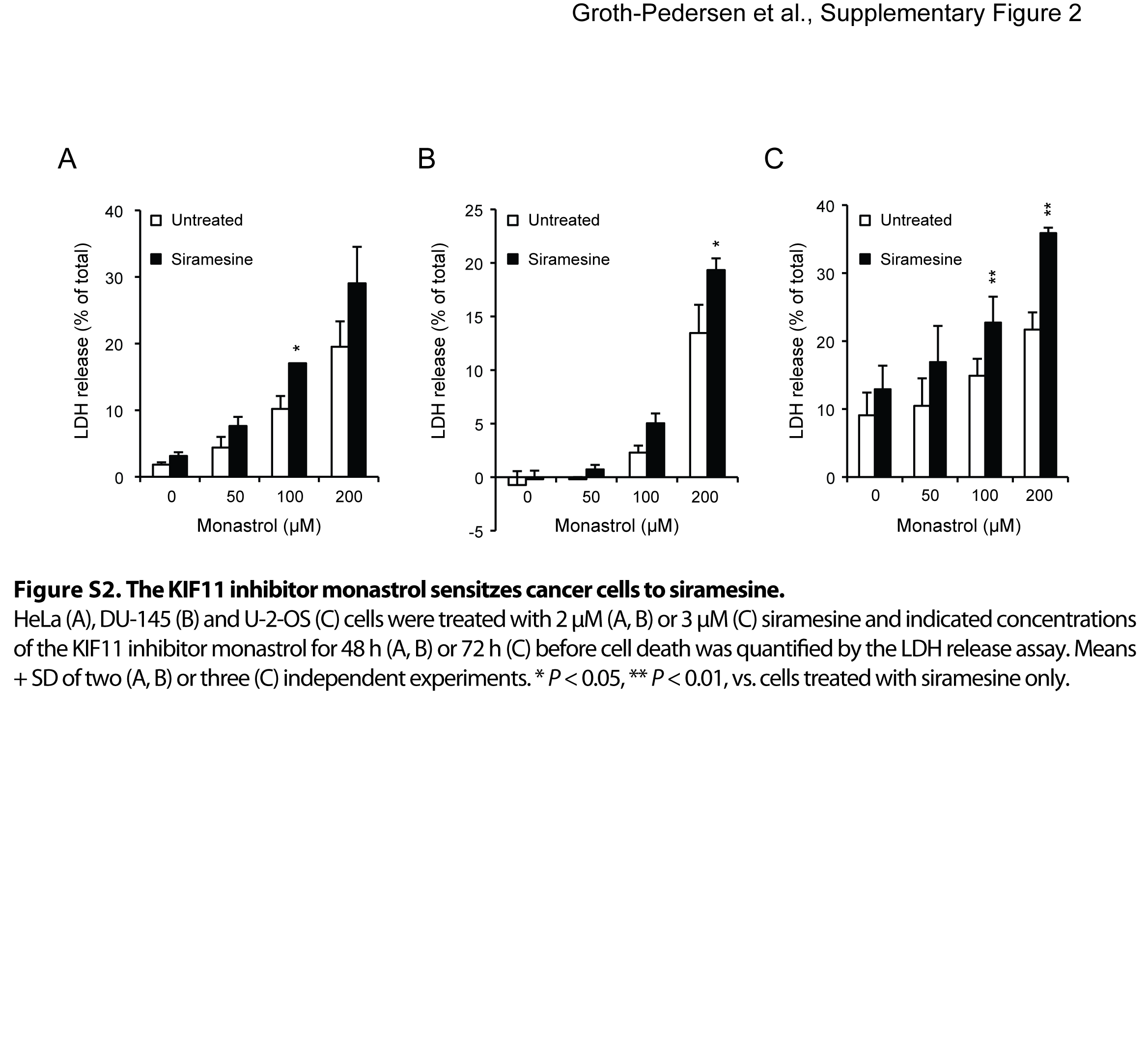

Supplement: Figure S2 — The KIF11 inhibitor monastrol sensitizes cancer cells to siramesine. (TIF) [file pone.0045381.s003.tif]
